# Supplementary material for: Citizens can help to map putative transmission sites for snail-borne diseases
Source: PLoS Negl Trop Dis. 2024 Apr 4;18(4):e0012062. doi: 10.1371/journal.pntd.0012062 (PMC11020946; doi:10.1371/journal.pntd.0012062)
Supplement: S1 Text — (PDF) [file pntd.0012062.s001.pdf]

S1 Text: The data collection protocol used by citizen scientists in the Kobo Collect mobile application

ATRAP: Citizen Researcher Protocol V3

* This is the start of the protocol, please make sure you are wearing your protective gear. Remember, safety first!

○ OK

ID

○ 1	○ 6	○ 11	○ 16	○ 21
○ 2	○ 7	○ 12	○ 17	○ 22
○ 3	○ 8	○ 13	○ 18	○ 23
○ 4	○ 9	○ 14	○ 19	○ 24
○ 5	○ 10	○ 15	○ 20	○ 25

Watercontactsite

Are you sampling this site today?

○ Yes

○ No

Why are you not sampling?

○ I have to attend a ceremony (funeral, wedding, ...)

○ Bad weather

○ Lack of transport

○ Household activites

○ Lack of compensation

○ Hostility from the community members

○ Lack of motivation

○ I am sick

○ A family member is sick

○ Lack of equipment

○ Other

f other, please specify:

____

Is it correct that you have a probe?

- ( ) YES
- ( ) NO

Enter the starting time

hh:mm

Select the date

yyyy-mm-dd

Take an overview photo of the water site

Haga clic aquí para subir el archivo. (<5MB)

Take a GPS point

latitude (x.y °)

longitude (x.y °)

altitude (m)

accuracy (m)

Has the water site been used this week?

- ( ) YES
- ( ) NO
- ( ) I don't know

Why was the water site not used this week?

- [ ] The water is too dirty
- [ ] The water flows too fast
- [ ] The water is not accessible
- [ ] The water level is too low
- [ ] There are dangerous animals
- [ ] There is NO water
- [ ] Other

If the water site was not accessible this week, please specify why:

If the water site was not used this week for other reasons, please specify.

Is the water site being used now?

- ○ YES
- ○ NO

What activities are going on at this moment?

- ☐ Drinking
- ☐ Swimming
- ☐ Bathing/washing
- ☐ Fishing
- ☐ Washing car
- ☐ Washing motorcycle
- ☐ Washing clothes
- ☐ Washing food
- ☐ Washing household utensils/tools
- ☐ Animal herding
- ☐ Wild animals are present
- ☐ Open defecation
- ☐ Open urination
- ☐ Fetching water
- ☐ Loading or unloading boats

Who is drinking the water?

- ☐ Man
- ☐ Woman
- ☐ Child

How many men are drinking from the water?

How many women are drinking from the water?

How many children are drinking from the water?

Who is drinking the water?

- ☐ Man
- ☐ Woman
- ☐ Child

How many men are drinking from the water?

____

How many women are drinking from the water?

____

How many children are drinking from the water?

____

Who is swimming in the water?

- [ ] Man
- [ ] Woman
- [ ] Child

How many men are swimming in the water?

____

How many women are swimming in the water?

____

How many children are swimming in the water?

____

Who is bathing in the water?

- [ ] Man
- [ ] Woman
- [ ] Child

How many men are bathing in the water?

____

How many women are bathing in the water?

____

How many children are bathing in the water?

____

Who is fishing in the water?

- [ ] Man
- [ ] Woman
- [ ] Child

How many men are fishing?

____

How many women are fishing?

____

How many children are fishing?

____

Who is washing car?

- [ ] Man
- [ ] Woman
- [ ] Child

How many men are washing cars?

How many women are washing cars?

How many children are washing cars?

Who is washing motorcycle?

- [ ] Man
- [ ] Woman
- [ ] Child

How many men are washing motorcycles?

How many women are washing motorcycles?

How many children are washing motorcycles?

Who is washing clothes?

- [ ] Man
- [ ] Woman
- [ ] Child

How many men are washing clothes?

How many women are washing clothes?

How many children are washing clothes?

Who is washing food?

- [ ] Man
- [ ] Woman
- [ ] Child

How many men are washing food?

How many women are washing food?

How many children are washing food?

____

Who is washing household utensils/tools?

- [ ] Man
- [ ] Woman
- [ ] Child

How many men are washing household utensils/tools?

____

How many women are washing household utensils/tools?

____

How many children are washing household utensils/tools?

____

Who is herding animals?

- [ ] Man
- [ ] Woman
- [ ] Child

How many men are herding animals?

____

How many women are herding animals?

____

How many children are herding animals?

____

Which domestic animals are present?

- [ ] Cattle
- [ ] Sheep
- [ ] Goats
- [ ] Pigs
- [ ] Chicken
- [ ] Dogs
- [ ] Others

If other domestic animals are present, please specify:

____

Which wild animals are present?

- [ ] Antelope
- [ ] Hippopotamus
- [ ] Monkey
- [ ] Wild hog
- [ ] Crocodile
- [ ] Baboon
- [ ] Elephant
- [ ] Bird
- [ ] Buffalo
- [ ] Others

If other wild animals are present, please specify:

____

The open defecation is done by whom?

- [ ] Man
- [ ] Woman
- [ ] Child

How many men are defecating in the open?

____

How many women are defecating in the open?

____

How many children are defecating in the open?

____

The open urination is done by whom?

- [ ] Man
- [ ] Woman
- [ ] Child

How many men are urinating in the open?

____

How many women are urinating in the open?

____

How many children are urinating in the open?

____

Who is fetching water?

- [ ] Man
- [ ] Woman
- [ ] Child

How many men are fetching water?

____

How many women are fetching water?

____

How many children are fetching water?

____

Who is loading or unloading boats?

- [ ] Man
- [ ] Woman
- [ ] Child

* Throw the thermometer in the water and let it sit there. Make sure the rope is fixed, so it does not float away. Then, continue the protocol.

○ OK

Do you have aqua test strips?

- [ ] Yes
- [ ] No

* Take out one test strip and dip it in the water for one second. Click "ok" after one minute.

○ OK

Place the test strip on the left side of the color chart (indicated in gray) and take a clear photograph.

Haga clic aquí para subir el archivo. (<5MB)

Take the thermometer out of the water and note the temperature.

____

* Turn on the probe by pressing the left button (on/mode), fill your container with water and insert the probe.

○ OK

What is the temperature? (if you could not take the measurement, please enter 0)

____

What is the pH? (if you could not take the measurement, please enter 0)

____

What is the μS? (if you could not take the measurement, please enter 0)

____

What is the ppm? (if you could not take the measurement, please enter 0)

____

* Put a few drops of storage liquid in the cap and close the probe.

○ OK

What is the time now?

hh:mm

* Scoop actively for snails for 30 minutes. Keep all snails that you find aside and COUNT the number of scoops you make. Click "ok" when you are done.

○ OK

You are done scooping. What is the time now?

hh:mm

How many scoops did you do?

Did you find any snails?

- Yes
- No

Place all snails on the scale paper and take a photograph.

Haga clic aquí para subir el archivo. (<5MB)

* Use the identification key to determine the different types of snails.

- OK

Please explain why you did not scoop:

What is the number of Biomphalaria specimens? (example shown below)

µ

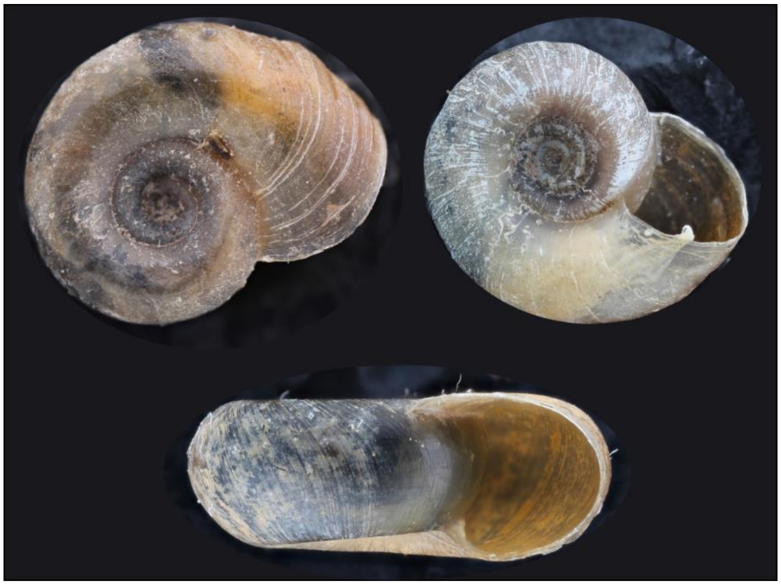

Place all Biomphalaria specimens on the scale paper and take a photograph.

Haga clic aquí para subir el archivo. (<5MB)

What is the number of Bulinus specimens? (example shown below)

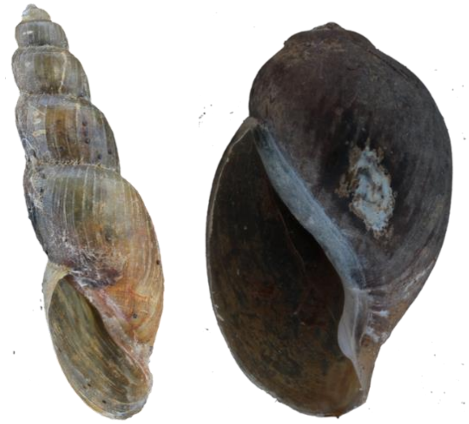

____

Place all Bulinus specimens on the scale paper and take a photograph.

Haga clic aquí para subir el archivo. (<5MB)

What is the number of Lymnea specimens? (example shown below)

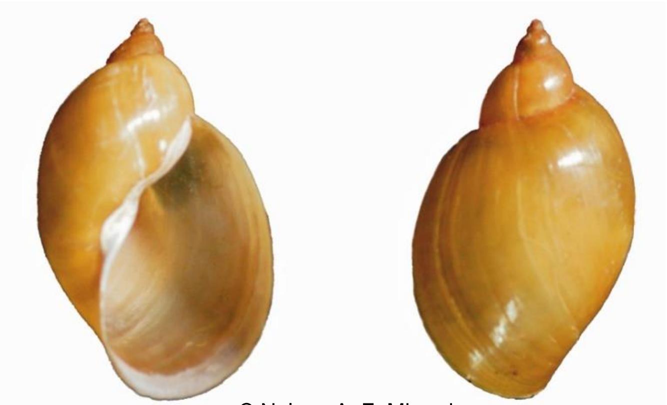

© Nelson A. F. Miranda

____

Place all Lymnea specimens on the scale paper and take a photograph.

Haga clic aquí para subir el archivo. (<5MB)

Did you find any other snails? (NO Biomphalaria, Bulinus or Lymnaea)

- YES
- NO

Place all other specimens on the scale paper and take a photograph.

Haga clic aquí para subir el archivo. (<5MB)

Enter the ending time

hh:mm
