# Supplementary material for: Citizens can help to map putative transmission sites for snail-borne diseases
Source: PLoS Negl Trop Dis. 2024 Apr 4;18(4):e0012062. doi: 10.1371/journal.pntd.0012062 (PMC11020946; doi:10.1371/journal.pntd.0012062)

S1 Fig**.** Site types used in the grouping of data collected by Citizen Scientists (CS) and the expert. Springs are underground water sources trapped by concrete walls to direct water through a metallic pipe with continuously flowing water, accessed by the communities. Stream sites are water contact sites with flowing surface water. Lake sites on Lake Albert were highly used water contact sites within fishing villages in Ndaiga Sub County and Ntoroko Town Council, on the South-Eastern side of the lake. Wetland sites consist of flooded areas for most of the year mostly characterised by rooted aquatic vegetation. All the photos were taken by the author (JT).

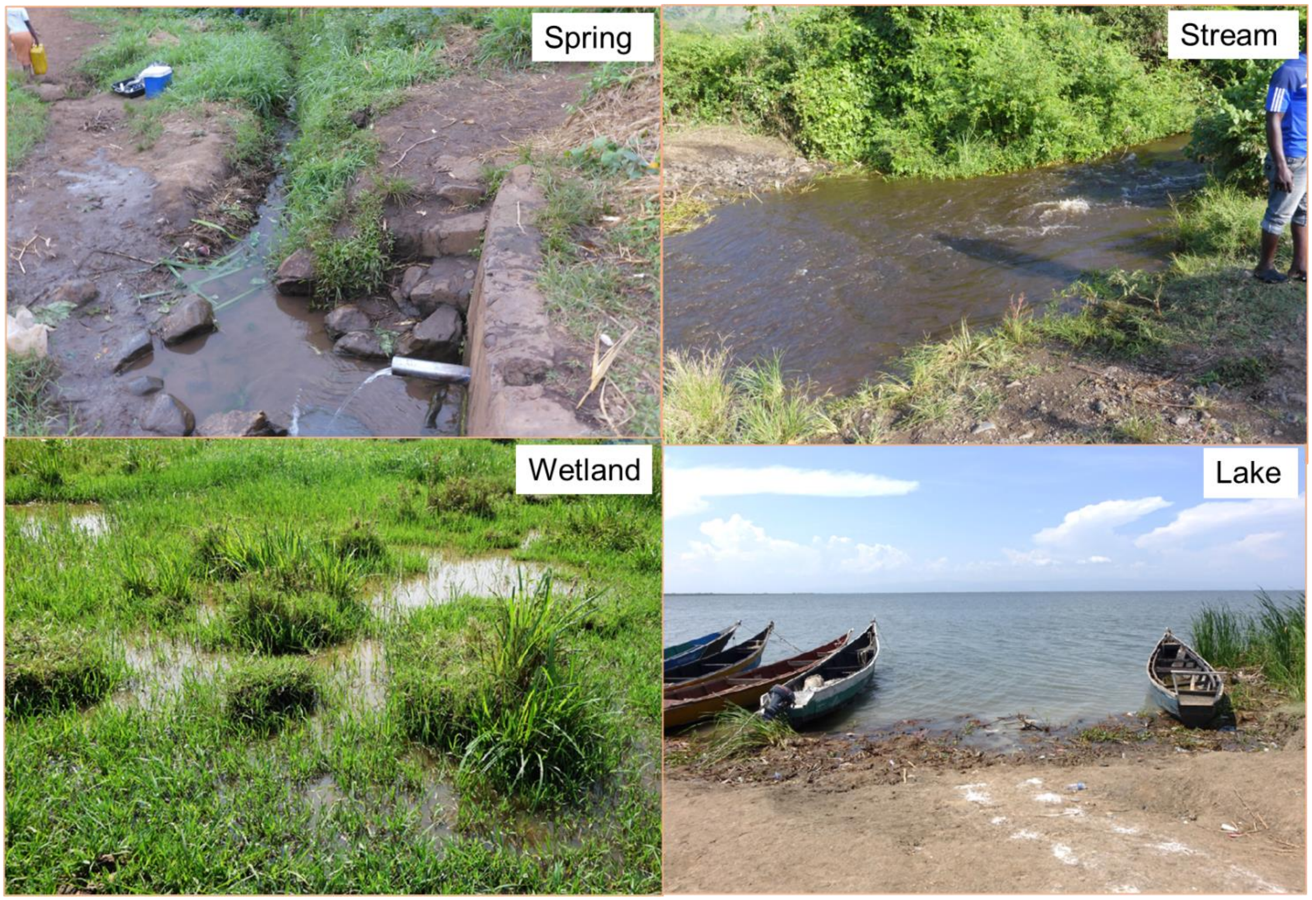

Supplement: S1 Fig — Springs are underground water sources trapped by concrete walls to direct water through a metallic pipe with continuously flowing water, accessed by the communities. Stream sites are water contact sites with flowing surface water. Lake sites on Lake Albert were highly used water contact sites within fishing villages in Ndaiga Sub County and Ntoroko Town Council, on the South-Eastern side of the lake. Wetland sites consist of flooded areas for most of the year mostly characterised by rooted aquatic vegetation. (PDF) [file pntd.0012062.s002.pdf]
