# Supplementary material for: Citizens can help to map putative transmission sites for snail-borne diseases
Source: PLoS Negl Trop Dis. 2024 Apr 4;18(4):e0012062. doi: 10.1371/journal.pntd.0012062 (PMC11020946; doi:10.1371/journal.pntd.0012062)
Supplement: S2 Fig — We explored the potential impact of snail removal without replacement by the expert. It was hypothesised that if snail removal impacted the extent of agreement between the expert and CS data, agreement would be higher if the CS sampled before the expert, more so on same date. Similarly, the effect of snail removal by the expert was expected to decrease with time due to migration. Thus, “Same” stands for the expert and CS having sampled a site on the same day, “Before” when the expert sampled before the CS and “After” when the expert sampled after the CS. If the sampling date difference was between one and three, it was considered “Close” (grey error bars) and if the difference was between four and six days, it was considered “Apart” (black error bars). Except for Biomphalaria, the difference in sampling days did not explain significant agreement/disagreement in snail presence/absence. Across all the genera and site types, the effect of snail removal by the expert was not significant (X2 (2) = (2.3, 2.1 & 2.5) p > 0.05, for Biomphalaria, Bulinus and Radix respectively) when the expert sampled before the CS. (PDF) [file pntd.0012062.s003.pdf]

S2 Fig. The effect of expert snail removal and sampling date difference with the CS for *Biomphalaria*, *Bulinus* and *Radix* snails at the different site types. We explored the potential impact of snail removal without replacement by the expert. It was hypothesised that if snail removal impacted the extent of agreement between the expert and CS data, agreement would be higher if the CS sampled before the expert, more so on same date. Similarly, the effect of snail removal by the expert was expected to decrease with time due to migration. Thus, “Same” stands for the expert and CS having sampled a site on the same day, “Before” when the expert sampled before the CS and “After” when the expert sampled after the CS. If the sampling date difference was between one and three, it was considered “Close” (grey error bars) and if the difference was between four and six days, it was considered “Apart” (black error bars). Except for *Biomphalaria*, the difference in sampling days did not explain significant agreement/disagreement in snail presence/absence. Across all the genera and site types, the effect of snail removal by the expert was not significant (X^2 (2) = (2.3, 2.1 & 2.5) $p > 0.05$, for *Biomphalaria, Bulinus* and *Radix* respectively) when the expert sampled before the CS.

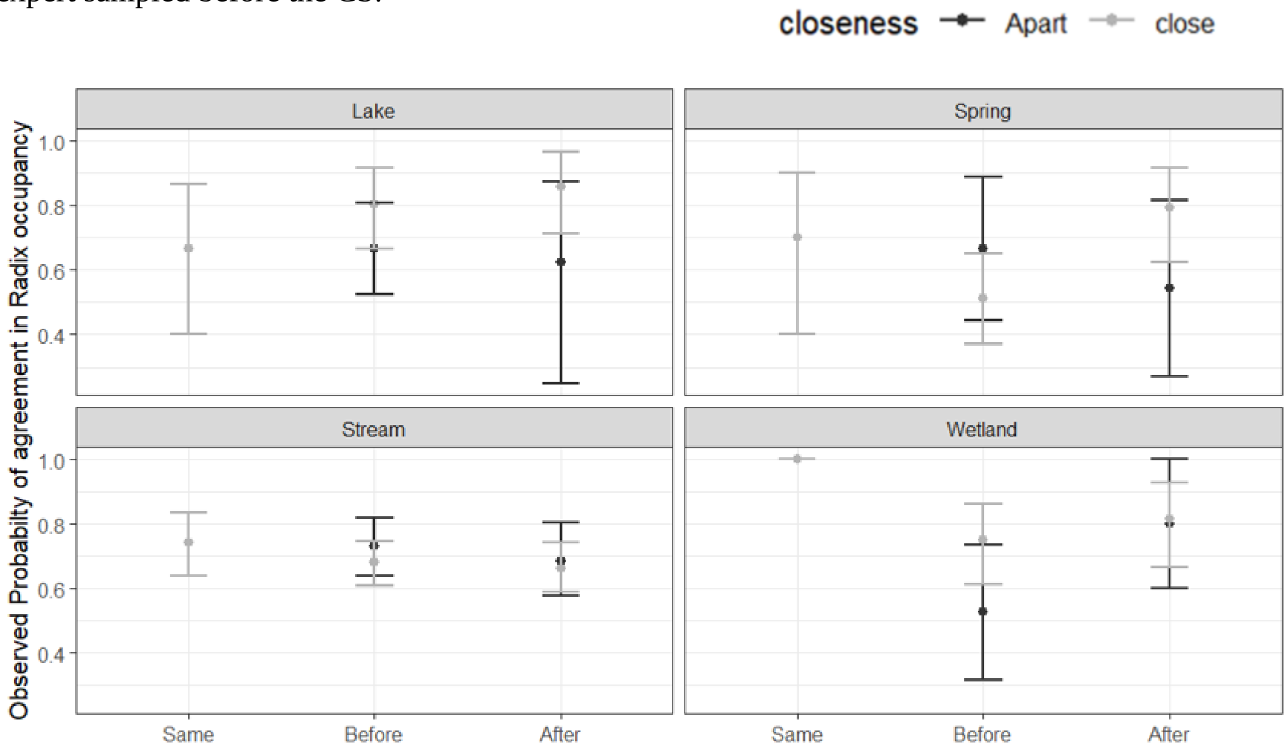

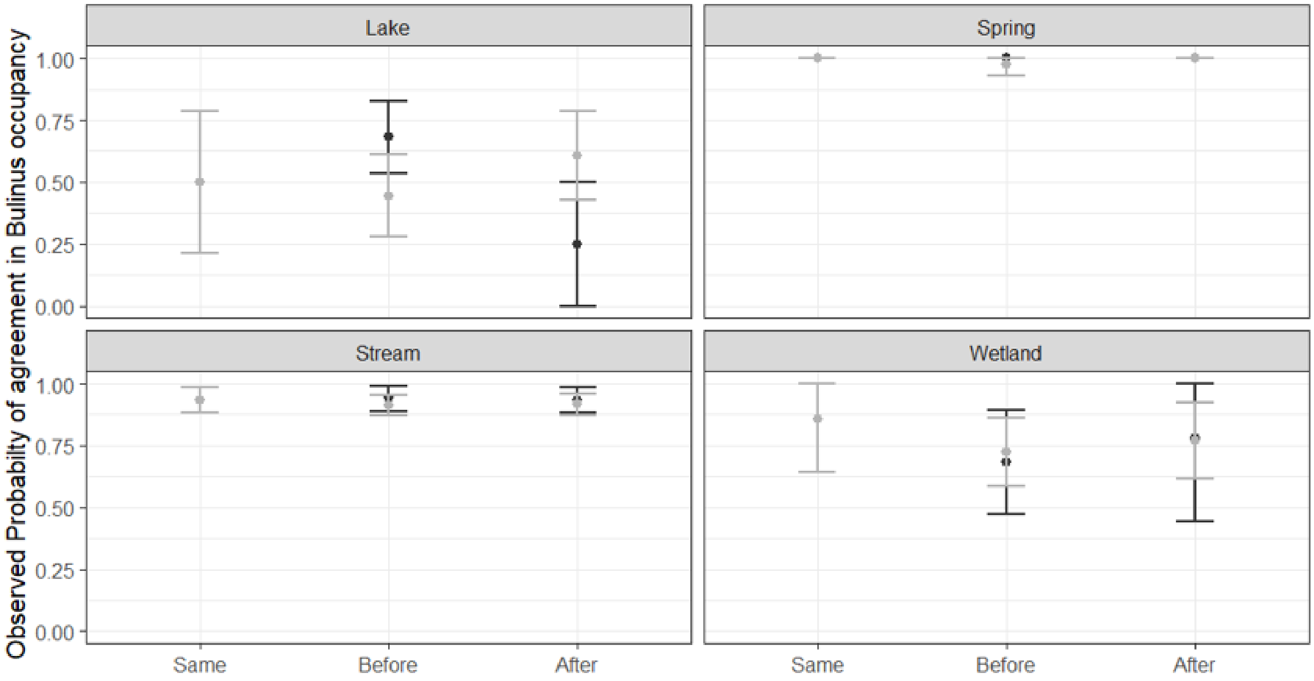
Lake
Spring
Stream
Wetland
Observed Probabilty of agreement in Bulinus occupancy
1.00
0.75
0.50
0.25
0.00
Same
Before
After

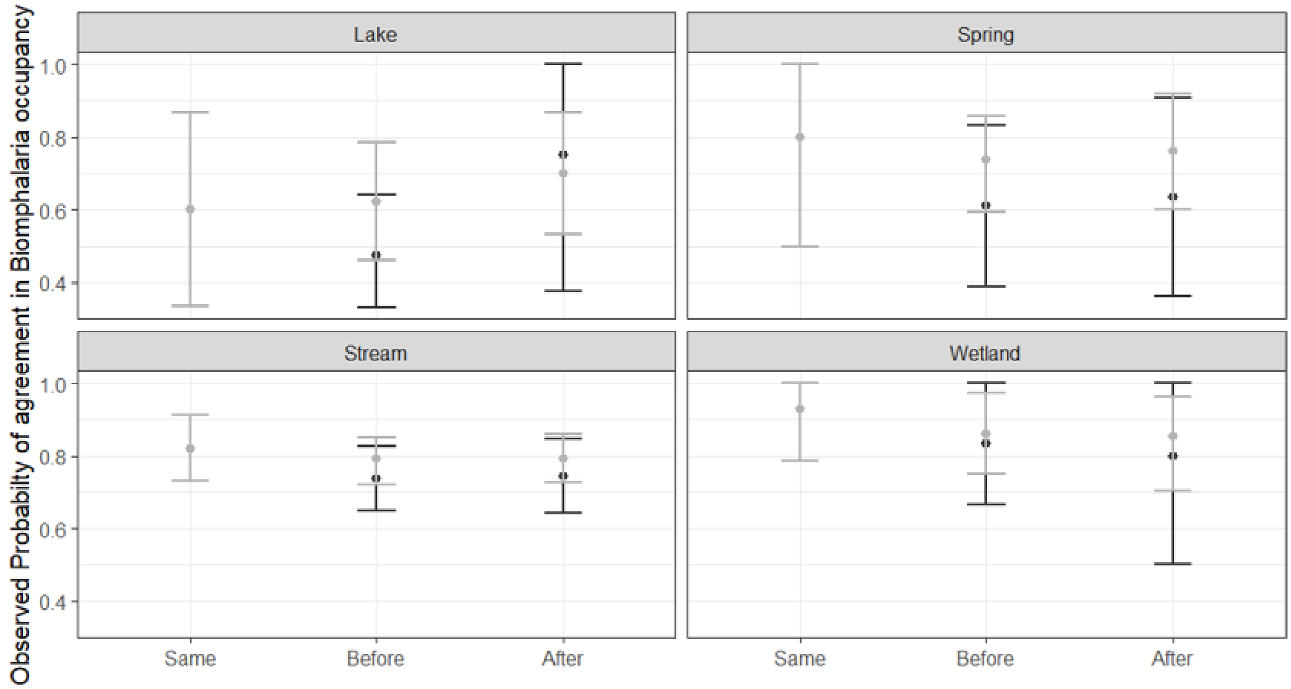
Lake
Spring
Stream
Wetland
Observed Probabilty of agreement in Biomphalaria occupancy
1.0
0.8
0.6
0.4
Same
Before
After
