# Supplementary material for: Citizens can help to map putative transmission sites for snail-borne diseases
Source: PLoS Negl Trop Dis. 2024 Apr 4;18(4):e0012062. doi: 10.1371/journal.pntd.0012062 (PMC11020946; doi:10.1371/journal.pntd.0012062)

S3 Fig. Observed probability of binary agreement (presence/absence) of *Biomphalaria* (A), *Bulinus* (B) and *Radix* (C) compared in the period when the expert removed snails from a site without replacement (16 months) with when the expert replaced the snails (4 months). For the three genera, the probabilities of agreement are statistically different with p<0.05. If snail removal changes snail population dynamics, the probability of agreement was expected to be lower when there was snail removal. However, except for *Radix* snails, the probability of was higher in the period when there was snail removal, contrary to our expectation. There was no clear pattern in agreement as a result of snail removal implying no clear influence of the snail removal by the expert on agreement in snail presence/absence.

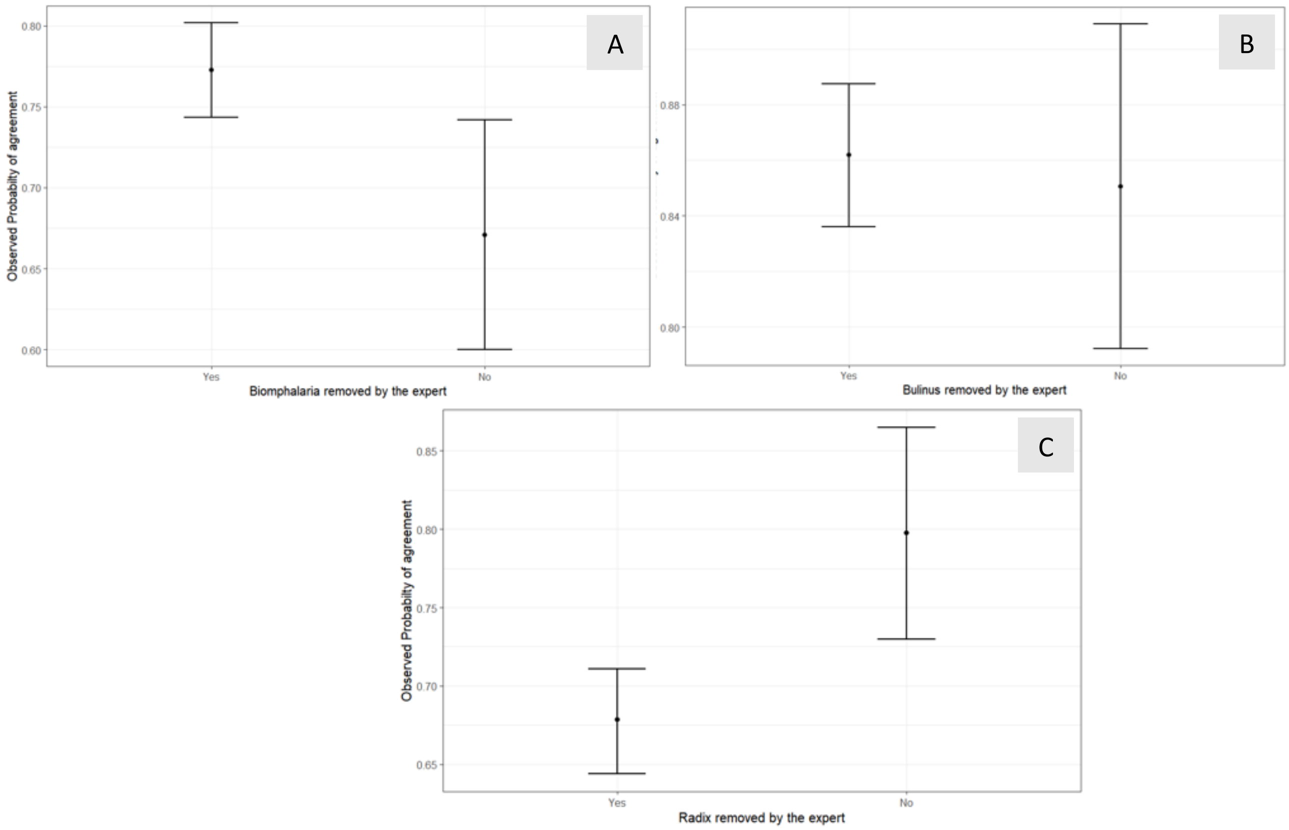

Supplement: S3 Fig — Observed probability of binary agreement (presence/absence) of Biomphalaria (A), Bulinus (B) and Radix (C) compared in the period when the expert removed snails from a site without replacement (16 months) with when the expert replaced the snails (4 months). For the three genera, the probabilities of agreement are statistically different with p<0.05. If snail removal changes snail population dynamics, the probability of agreement was expected to be lower when there was snail removal. However, except for Radix snails, the probability of was higher in the period when there was snail removal, contrary to our expectation. There was no clear pattern in agreement as a result of snail removal implying no clear influence of the snail removal by the expert on agreement in snail presence/absence. (PDF) [file pntd.0012062.s004.pdf]
