# Supplementary material for: Citizens can help to map putative transmission sites for snail-borne diseases
Source: PLoS Negl Trop Dis. 2024 Apr 4;18(4):e0012062. doi: 10.1371/journal.pntd.0012062 (PMC11020946; doi:10.1371/journal.pntd.0012062)

S4 Fig. Structure of the nested random effect implemented in the linear mixed-effect model. ID is the CS ID, loc is the sampling site/location.

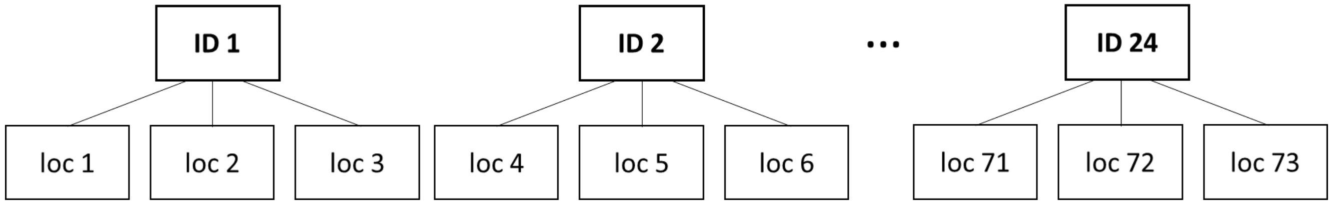

Supplement: S4 Fig — ID is the CS ID, loc is the sampling site/location. (PDF) [file pntd.0012062.s005.pdf]
