# Supplementary material for: Citizens can help to map putative transmission sites for snail-borne diseases
Source: PLoS Negl Trop Dis. 2024 Apr 4;18(4):e0012062. doi: 10.1371/journal.pntd.0012062 (PMC11020946; doi:10.1371/journal.pntd.0012062)

S5 Fig. Map of the study area showing total abundance (expert data 06/2020 – 04/2022) of the studied snail genera in each water contact site, black points indicate locations without snails. The base map was adopted from the OpenStreetMap database (https://www.openstreetmap.org/#map=13/1.0212/30.5998) in QGIS v3.4.4

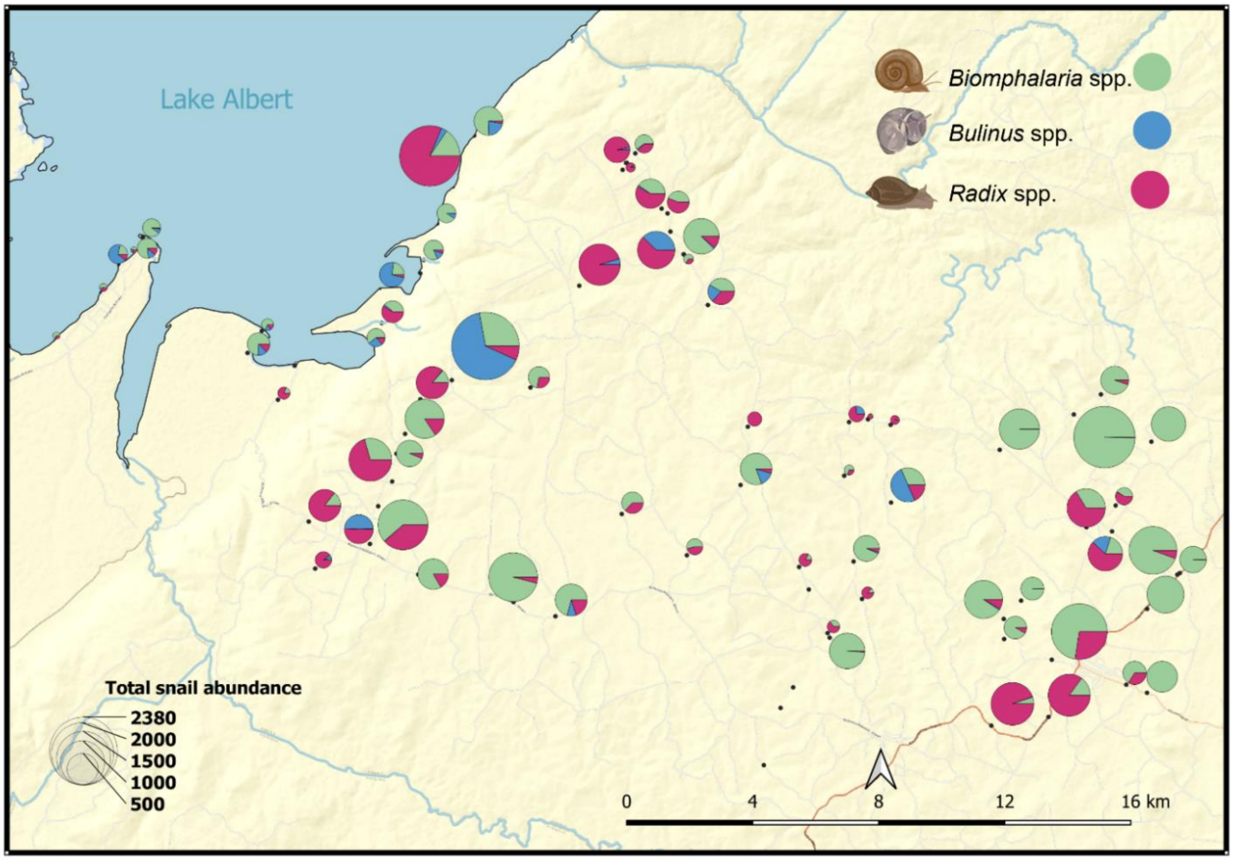

Supplement: S5 Fig — (PDF) [file pntd.0012062.s006.pdf]
