# Supplementary material for: Citizens can help to map putative transmission sites for snail-borne diseases
Source: PLoS Negl Trop Dis. 2024 Apr 4;18(4):e0012062. doi: 10.1371/journal.pntd.0012062 (PMC11020946; doi:10.1371/journal.pntd.0012062)

S6 Fig. Observed probability of binary agreement with time between the citizen scientists and the expert in *Radix* sp. presence/absence with time.

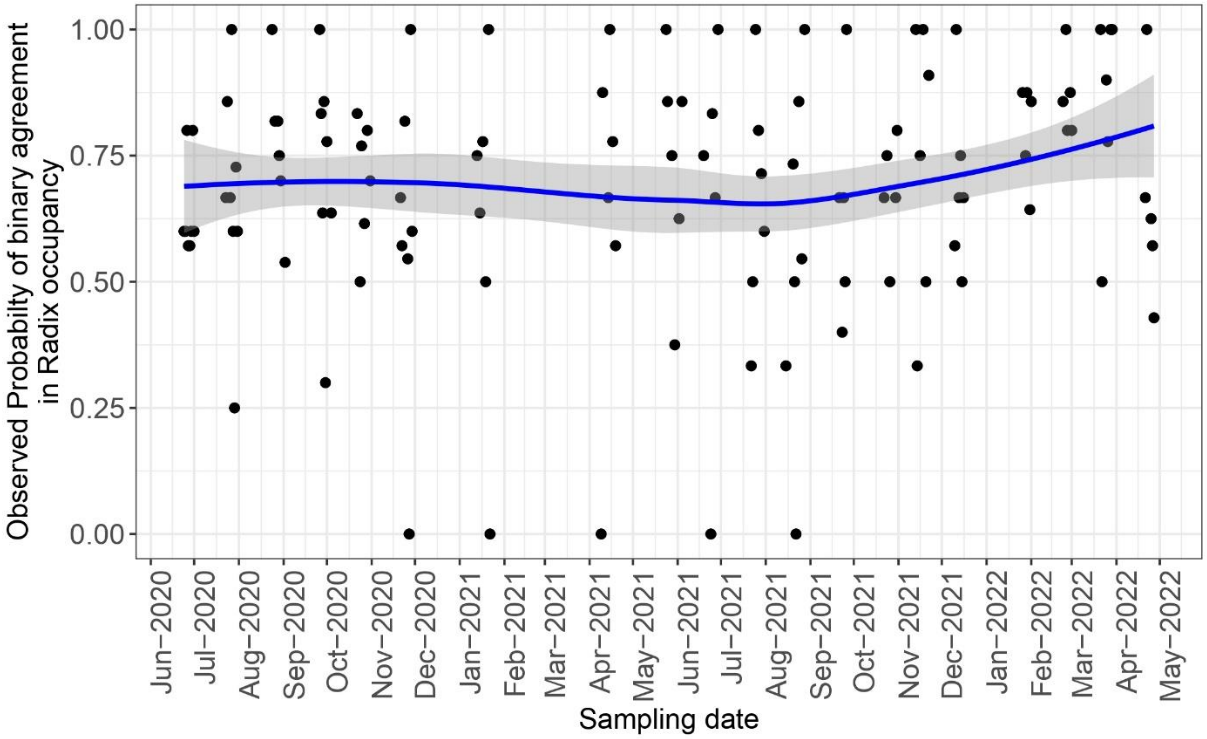

Supplement: S6 Fig — (PDF) [file pntd.0012062.s007.pdf]
