# Supplementary material for: Citizens can help to map putative transmission sites for snail-borne diseases
Source: PLoS Negl Trop Dis. 2024 Apr 4;18(4):e0012062. doi: 10.1371/journal.pntd.0012062 (PMC11020946; doi:10.1371/journal.pntd.0012062)

S7 Fig. Observed variation in the degree of agreement of each citizen scientists with the expert in the detection of presence/absence of *Biomphalaria*, *Bulinus* and *Radix* snails. We observed variations in agreement for the same citizen scientist when sampling different site types. The citizen scientist IDs were sorted from lowest to highest mean agreement with the expert for each snail genus.

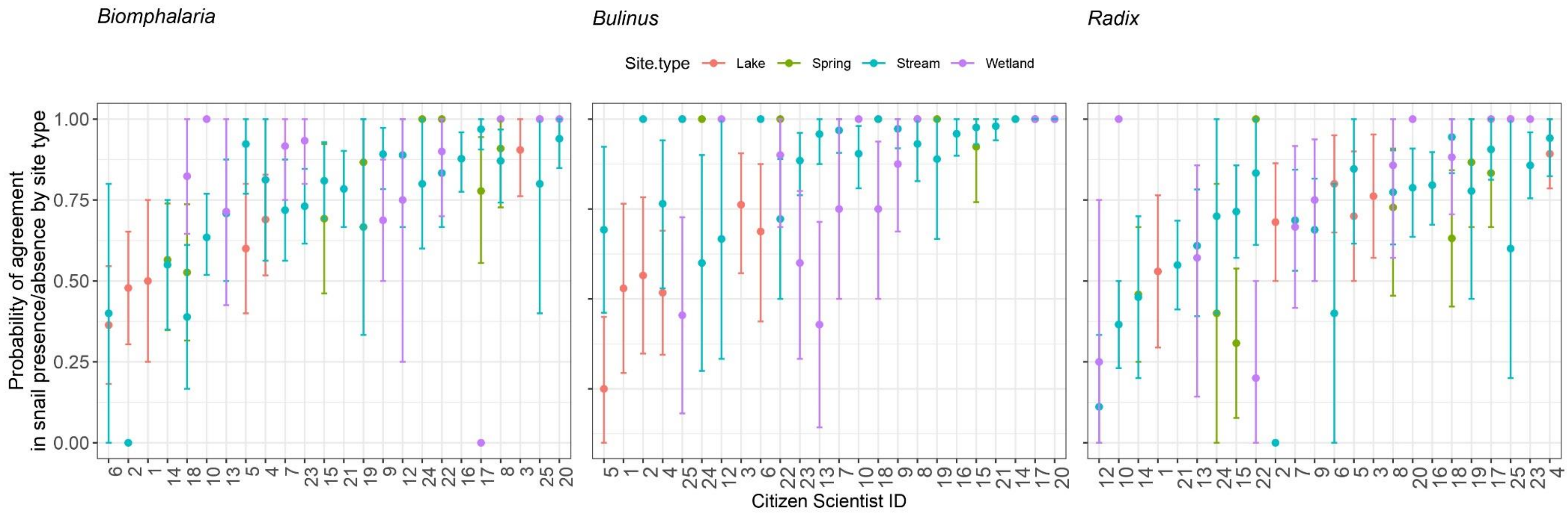

Supplement: S7 Fig — We observed variations in agreement for the same citizen scientist when sampling different site types. The citizen scientist IDs were sorted from lowest to highest mean agreement with the expert for each snail genus. (PDF) [file pntd.0012062.s008.pdf]
