# Supplementary material for: Citizens can help to map putative transmission sites for snail-borne diseases
Source: PLoS Negl Trop Dis. 2024 Apr 4;18(4):e0012062. doi: 10.1371/journal.pntd.0012062 (PMC11020946; doi:10.1371/journal.pntd.0012062)

**S8 Fig**. Analysis including cumulative abundance reported by the citizen scientists (dotted line) and the expert (full lines) per month (left panel). The differences between the graphs (Fig 3A – main text) are due to the number of points that are being considered (1037 points for each snail genera, in contrast with 911 paired reports on *Biomphalaria* spp., 900 on *Bulinus* spp. and 905 on *Radix* sp. from the original analysis) because originally, we only considered points that were apart +/- 7 days. The false positives increased, but also the agreement (TP + TN) increased for *Radix* by around 10%. [Snail icons created with BioRender.com]

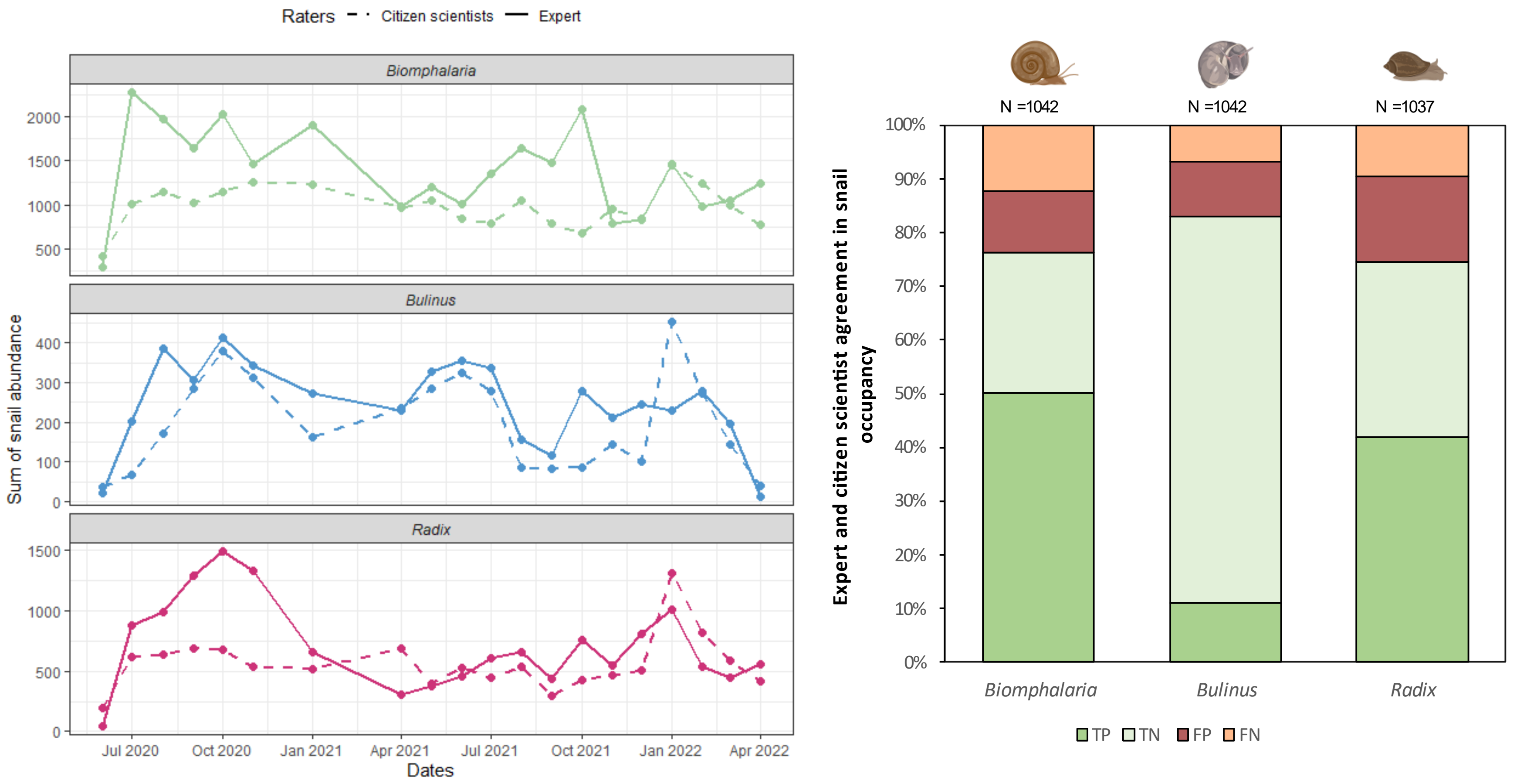

Supplement: S8 Fig — Analysis including cumulative abundance reported by the citizen scientists (dotted line) and the expert (full lines) per month (left panel). The differences between the graphs (Fig 3A–main text) are due to the number of points that are being considered (1037 points for each snail genera, in contrast with 911 paired reports on Biomphalaria spp., 900 on Bulinus spp. and 905 on Radix sp. from the original analysis) because originally, we only considered points that were apart +/- 7 days. The false positives increased, but also the agreement (TP + TN) increased for Radix by around 10%. (PDF) [file pntd.0012062.s009.pdf]
