# Supplementary figures and images for: Citizens can help to map putative transmission sites for snail-borne diseases
Source: PLoS Negl Trop Dis. 2024 Apr 4;18(4):e0012062. doi: 10.1371/journal.pntd.0012062 (PMC11020946; doi:10.1371/journal.pntd.0012062)

**S9 Fig.** Values of consistency and numerical per time step (month) for all the snail genera studied

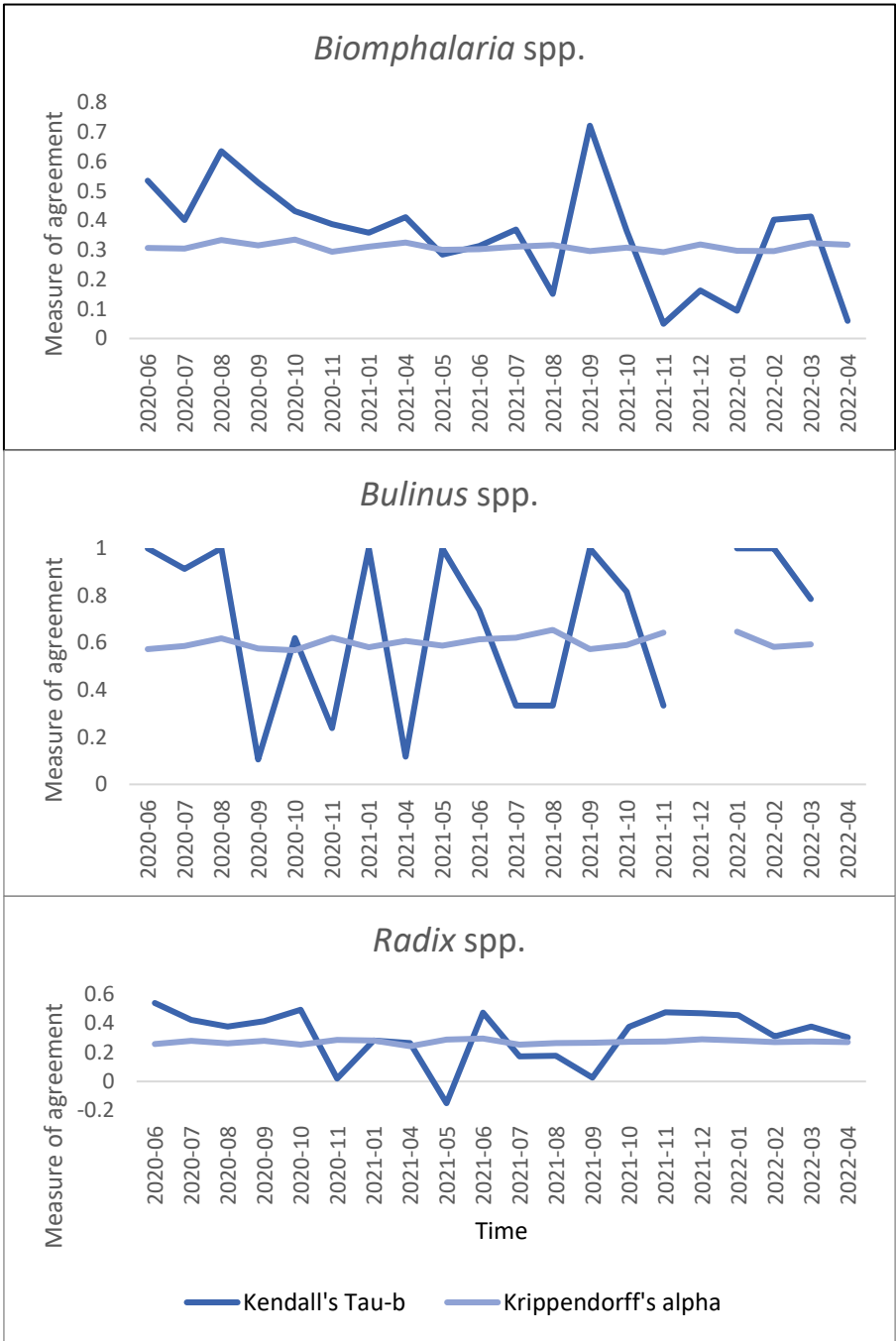

Supplement: S9 Fig — (PDF) [file pntd.0012062.s010.pdf]
