# Supplementary material for: Citizens can help to map putative transmission sites for snail-borne diseases
Source: PLoS Negl Trop Dis. 2024 Apr 4;18(4):e0012062. doi: 10.1371/journal.pntd.0012062 (PMC11020946; doi:10.1371/journal.pntd.0012062)
Supplement: S1 Table — (PDF) [file pntd.0012062.s011.pdf]

S1 Table. Variables used in the comparison of citizen scientists' and expert-collected snail data

Variable	Type	Levels
Citizen researcher’s ID	Nominal	1-25 (ID 11 excluded)
Site name	Nominal	73 unique names
Site type	Nominal	Stream, wetland, lake, or spring
Sampling date difference	Numerical	0 – 7
Snail species	Nominal	*Biomphalaria*, *Bulinus* & *Radix* species
Number of snails	Numerical	0 – 282 per 30 minutes of sampling
