# Supplementary material for: Citizens can help to map putative transmission sites for snail-borne diseases
Source: PLoS Negl Trop Dis. 2024 Apr 4;18(4):e0012062. doi: 10.1371/journal.pntd.0012062 (PMC11020946; doi:10.1371/journal.pntd.0012062)
Supplement: S2 Table — Ref. stands for reference, CI for confidence interval, and significant values are in bold. (PDF) [file pntd.0012062.s012.pdf]

S2 Table. *Biomphalaria*, *Bulinus* and *Radix* predictors of agreement in snail presence/absence between the citizen scientists and the expert, and their metrics. Ref. stands for reference, CI for confidence interval, and significant values are in bold.

			**Agreement**			
**Snail genus**	**Predictors, x_{ij}**	Effect size, β (95% CI)	Odds ratios (OR)	95% CI		*p*
				Lower	upper	
Biomphalaria	Intercept	0.57 (-0.04, 1.18)	1.76	0.96	3.25	0.068
	Snail abundance	0.02 (0.008, 0.02)	1.02	1.01	1.02	**<0.001**
	Sampling date difference	-0.10 (-0.18, -0.02)	0.91	0.83	0.98	**0.020**
	Site type (Ref. Lake)					
	Spring	0.49 (-0.38, 1.36)	1.63	0.68	3.89	0.272
	Stream	0.84 (0.22, 1.47)	2.33	1.24	4.35	**0.008**
	Wetland	1.46 (0.54, 2.38)	4.30	1.72	10.76	**0.002**
Bulinus	Intercept	0.39 (-0.33, 1.12)	1.48	0.72	3.05	0.284
	Site type (Ref. Lake)					
	Spring	4.65 (2.37, 6.93)	104.66	10.72	1021.46	**<0.001**
	Stream	2.47 (1.59, 3.35)	11.83	4.93	28.39	**<0.001**
	Wetland	1.21 (0.11, 2.32)	3.36	1.11	10.17	**0.032**
Radix	Intercept	0.85 (0.49, 1.20)	2.33	1.63	3.33	**<0.001**
	Snail abundance	0.0085 (0.00078, 0.02)	1.01	1.00	1.02	**0.031**
