# Supplementary material for: Citizens can help to map putative transmission sites for snail-borne diseases
Source: PLoS Negl Trop Dis. 2024 Apr 4;18(4):e0012062. doi: 10.1371/journal.pntd.0012062 (PMC11020946; doi:10.1371/journal.pntd.0012062)
Supplement: S3 Table — (PDF) [file pntd.0012062.s013.pdf]

We analysed two cases, case (a) when snail abundance reported by expert > snail abundance reported by CS, and we observed that for *Biomphalaria* and *Radix* snails the predictor: scaled date is statistically significant ($p<0.05$) with coefficients with values close to zero (Table S3).

**S3 Table:** Generalized linear mixed models output – case A

| | *Biomphalaria* spp**.** | | | *Radix* sp**.** | | |
|---|---|---|---|---|---|---|
| ***Predictors*** | ***Estimates*** | ***CI*** | ***p*** | ***Estimates*** | ***CI*** | ***p*** |
| (Intercept) | 2.59 | 2.01 – 3.18 | **<0.001** | 1.52 | 0.97 – 2.07 | **<0.001** |
| Sampling date difference | 0.01 | -0.04 – 0.06 | 0.667 | -0.01 | -0.05 – 0.04 | 0.788 |
| Date | 0.00 | 0.00 – 0.00 | **0.001** | 0.00 | 0.00 – 0.00 | **0.003** |
| Site type (ref. Lake) | | | | | | |
| Spring | 0.07 | -0.66 – 0.81 | 0.841 | 0.41 | -0.26 – 1.08 | 0.231 |
| Stream | -0.38 | -0.93 – 0.18 | 0.182 | 0.41 | -0.11 – 0.94 | 0.121 |
| Wetland | -0.79 | -1.53 – -0.05 | **0.036** | 0.95 | 0.33 – 1.57 | **0.003** |
| Observations | 470 | | | 440 | | |
| Marginal $R^2$ | 0.051 | | | 0.055 | | |
| Conditional $R^2$ | 0.343 | | | 0.378 | | |
