# Supplementary material for: Citizens can help to map putative transmission sites for snail-borne diseases
Source: PLoS Negl Trop Dis. 2024 Apr 4;18(4):e0012062. doi: 10.1371/journal.pntd.0012062 (PMC11020946; doi:10.1371/journal.pntd.0012062)
Supplement: S4 Table — (PDF) [file pntd.0012062.s014.pdf]

In the case of *Bulinus* spp., the result of the GLMM was a singular fit related to overfitting due to the complex random effect structure. Since the random effect structure is a characteristic of our sampling design, this configuration could not be altered, hence we did not include the results of *Bulinus* spp. in S4 Table.

For case (b) snail abundance reported expert < CS snails, we did not find any variable with statistical significance ($p<0.05$) (S4 Table). In addition, the results for *Radix* sp., indicated a singular fit related to overfitting due to the complex random effect structure.

S4 Table: Generalized linear mixed models output – case B

	Biomphalaria spp.			*Bulinus* spp.		
Predictors	Estimates	CI	*p*	Estimates	CI	*p*
(Intercept)	1.84	1.24 – 2.43	**<0.001**	0.29	-0.84 – 1.41	0.61
Sampling date difference	-0.02	-0.08 – 0.04	0.568	0.12	-0.04 – 0.28	0.13
Date	0	-0.00 – 0.00	0.74	0	-0.00 – 0.00	0.39
Site type (ref. Lake)						
Spring	0.44	-0.33 – 1.20	0.261	0.95	-0.63 – 2.53	0.23
Stream	-0.01	-0.58 – 0.56	0.975	0.72	-0.25 – 1.69	0.14
Wetland	-0.25	-1.13 – 0.63	0.57	0.23	-0.98 – 1.43	0.7
Observations			165			35
Marginal R^2	0.032			0.141		
Conditional R^2	0.403			0.512		
