# Supplementary material for: Citizens can help to map putative transmission sites for snail-borne diseases
Source: PLoS Negl Trop Dis. 2024 Apr 4;18(4):e0012062. doi: 10.1371/journal.pntd.0012062 (PMC11020946; doi:10.1371/journal.pntd.0012062)
Supplement: S5 Table — (PDF) [file pntd.0012062.s015.pdf]

S5 Table. Analysis including cumulative abundance per month (CS) / Abundance

Consistency (Kendall Tau-b) and numerical agreement (Krippendorff's alpha)

Site	*Biomphalaria* spp.				*Bulinus* spp.				*Radix* sp.			
		Cons.		Agree.		Cons.		Agree.		Cons.		Agree.
	N	K. Tau-b	*p*	K. α	N	K. Tau-b	*p*	K. α	N	K. Tau-b	*p*	K. α
Lake	37	0.10	0.39	0.14	16	0.24	0.24	0.11	25	0.22	0.15	0.16
Spring	52	**0.37**	**<0.01**	0.39	14	**0.54**	**0.01**	0.35	38	**0.17**	0.15	0.11
Stream	401	**0.35**	**0.01**	0.32	50	**0.45**	**<0.01**	0.45	287	**0.37**	**<0.01**	0.36
Wetland	33	**0.34**	**<0.01**	0.45	36	**0.52**	**<0.01**	0.47	85	**0.29**	**<0.01**	0.25

Only the statistically significant values are in bold (Kendall Tau-b) In general, the consistency is lower with the new arrangement, but the agreement increases (this is something expected since always the expert was reporting more than the citizens and now the values are closer).
