# Supplementary material for: Citizens can help to map putative transmission sites for snail-borne diseases
Source: PLoS Negl Trop Dis. 2024 Apr 4;18(4):e0012062. doi: 10.1371/journal.pntd.0012062 (PMC11020946; doi:10.1371/journal.pntd.0012062)
Supplement: S6 Table — For the expert, two scenarios are considered: when one expert samples each site once a month as in our approach, and if the expert were to sample each site four times a month like the citizen scientists in this study. (PDF) [file pntd.0012062.s016.pdf]

S6 Table. Summary of cost of data collection by the citizen scientist compared to the expert. For the expert, two scenarios are considered: when one expert samples each site once a month as in our approach, and if the expert were to sample each site four times a month like the citizen scientists in this study.

	Item	Units	Unit cost (UGX)	20 months (UGX)	20 months (1€ = 4000 UGX)
Expert	Fuel	167	6,000	20,040,000	5,010
	Vehicle hire	12	300,000	72,000,000	18,000
	Per diem for the expert	12	150,000	36,000,000	9,000
	Per diem for the driver	12	150,000	36,000,000	9,000
	Scoop net	1	100,000	100,000	25
	Monthly facilitation lumpsum‡	1	720,000	14,400,000	3600
	Gum boots	2	50,000	100,000	25
	Disposable gloves	2	30,000	1,200,000	300
	TOTAL*1 sampling time per site per month			178,640,000	44,660
	TOTAL*4 sampling times per site per month			714,560,000	178,640
Citizen scientists	Monthly compensation	24	75,000	36,000,000	9,000
	Smartphones	24	800,000	19,200,000	4,800
	Scoop nets	24	100,000	2,400,000	600
	Gum boots	24	50,000	1,200,000	300
	Disposable gloves	24	30,000	14,400,000	1,200
	Data validation cost	-	-	-	210
	Annual trainings on data collection and safety	3	-	29,460,000	7,365
	TOTAL*4 sampling times per site per month			102,660,000	23,475

‡ Note that the cost for the monthly facilitation of the expert is based on conservative estimates of half of the stipend of a PhD student dedicating 14 days a month. The cost would be much higher if a hired expert with a salary is considered.
