# Supplementary material for: Citizens can help to map putative transmission sites for snail-borne diseases
Source: PLoS Negl Trop Dis. 2024 Apr 4;18(4):e0012062. doi: 10.1371/journal.pntd.0012062 (PMC11020946; doi:10.1371/journal.pntd.0012062)
Supplement: Attachment — Submitted filename: PNTD-D-23-00886.pdf [file pntd.0012062.s017.pdf]

PLOS Neglected Tropical Diseases

Citizens can help to map putative transmission sites for snail-borne diseases

--Manuscript Draft--

**Manuscript Number:**	PNTD-D-23-00886
**Full Title:**	Citizens can help to map putative transmission sites for snail-borne diseases
**Short Title:**	Citizen science and snail-borne disease monitoring
**Article Type:**	Research Article
**Keywords:**	Citizen science; data validation; inter-rater reliability; schistosomiasis; fasciolosis; snail control

Abstract:

Introduction
Schistosomiasis is a snail-borne disease that affects over 200 million people globally, 90% living in Sub-Saharan Africa. Similarly, fasciolosis - an emerging zoonotic snail-borne disease, causes significant damage to human health and to the livestock industry. However, shortage of snail experts and data hinders implementing the WHO recommendations on snail control to supplement drug treatment towards disease elimination. Therefore, we adopted a citizen science approach to snail monitoring of <i>Biomphalaria</i>, <i>Bulinus</i> and <i>Radix</i> intermediate hosts of snail-borne diseases.
Materials and methods
Snail data was collected weekly by 25 trained citizen scientists (CSs) at 76 sites around southern Lake Albert (Uganda) for 20 months. At each site, snails were searched for 30 minutes, sorted, target snail hosts identified to genus level, counted and data submitted through a smartphone application. The quality of this data was assessed by comparing it to monthly data collected by an ‘expert’ malacologist using the same sampling protocol. Generalised binomial logistic and linear mixed-effects models were used to analyse the variables for agreement between the CSs and expert
Findings
The binary agreement in presence/absence of <i>Biomphalaria</i>, <i>Bulinus</i> and <i>Radix</i> snails reported by the expert and CSs ranged between 70% and 86% (900 reports) with an average of 17% false negatives (sites wrongly defined as snail-free). The agreement for <i>Biomphalaria</i> and<i>Radix</i> increased with snail abundance, while false negatives decreased when the number of snails collected by citizens were aggregated per month. Site type significantly predicted binary agreement, which was lowest at lake sites (55%) and highest at spring sites (99%) with variations across genera. Similar temporal trends in snail abundance were recorded despite the expert reporting higher abundance. However, the relative abundance was consistent across site types. The match between the sites with highest <i>Biomphalaria</i> spp. abundance identified by CSs and expert was consistently high (~84.1%) and increased over time.
Conclusions and recommendations
Our results demonstrate the potential of citizen science to map putative schistosomiasis transmission sites. We therefore argue that this inclusive, powerful and cost-effective approach can be more sustainable than top-down monitoring and intervention campaigns.

**Additional Information:**	
**Question**	**Response**
**Government Employee**<br><br>Are you or any of the contributing authors an employee of the United States government?<br><br>Manuscripts authored by one or more US	No - No authors are employees of the U.S. government.

Powered by Editorial Manager® and ProduXion Manager® from Aries Systems Corporation

Government employees are not copyrighted, but are licensed under a CC0 Public Domain Dedication, which allows unlimited distribution and reuse of the article for any lawful purpose. This is a legal requirement for US Government employees.<br><br>This will be typeset if the manuscript is accepted for publication.	
**Financial Disclosure**<br><br>Enter a financial disclosure statement that describes the sources of funding for the work included in this submission. Review the submission guidelines for detailed requirements. View published research articles from *PLOS Neglected Tropical Diseases* for specific examples.<br><br>This statement is required for submission and **will appear in the published article** if the submission is accepted. Please make sure it is accurate.<br><br>**Funded studies**<br>Enter a statement with the following details:<br>• Initials of the authors who received each award<br>• Grant numbers awarded to each author<br>• The full name of each funder<br>• URL of each funder website<br>• Did the sponsors or funders play any role in the study design, data collection and analysis, decision to publish, or preparation of the manuscript?<br><br>Did you receive funding for this work?	Yes
Please add funding details.<br>as follow-up to "**Financial Disclosure**<br><br>Enter a financial disclosure statement that describes the sources of funding for the work included in this submission. Review the submission guidelines for detailed requirements. View published research articles from *PLOS Neglected Tropical*	This research was financed by the Action Towards Reducing Aquatic snail-borne Parasitic diseases (ATRAP) project of the Development Cooperation program of the Royal Museum for Central Africa and Mbarara University of Science and Technology with support of the Directorate-General Development Cooperation and Humanitarian Aid.

Diseases for specific examples.<br><br>This statement is required for submission and **will appear in the published article** if the submission is accepted. Please make sure it is accurate.<br><br>**Funded studies**<br>Enter a statement with the following details:<br>• Initials of the authors who received each award<br>• Grant numbers awarded to each author<br>• The full name of each funder<br>• URL of each funder website<br>• Did the sponsors or funders play any role in the study design, data collection and analysis, decision to publish, or preparation of the manuscript?<br><br>Did you receive funding for this work?"	
Please select the country of your main research funder (please select carefully as in some cases this is used in fee calculation).<br>as follow-up to "**Financial Disclosure**<br><br>Enter a financial disclosure statement that describes the sources of funding for the work included in this submission. Review the submission guidelines for detailed requirements. View published research articles from *PLOS Neglected Tropical Diseases* for specific examples.<br><br>This statement is required for submission and **will appear in the published article** if the submission is accepted. Please make sure it is accurate.	BELGIUM - BE

Powered by Editorial Manager® and ProduXion Manager® from Aries Systems Corporation

**Funded studies**<br>Enter a statement with the following details:<br>• Initials of the authors who received each award<br>• Grant numbers awarded to each author<br>• The full name of each funder<br>• URL of each funder website<br>• Did the sponsors or funders play any role in the study design, data collection and analysis, decision to publish, or preparation of the manuscript?<br><br>Did you receive funding for this work?"	
**Competing Interests**<br><br>On behalf of all authors, disclose any competing interests that could be perceived to bias this work.<br><br>This statement will be typeset if the manuscript is accepted for publication.<br><br>Review the instructions link below and PLOS NTDs' competing interests policy to determine what information must be disclosed at submission.	The authors have no competing interests to declare
**Data Availability**<br><br>Provide a **Data Availability Statement** in the box below. This statement should detail where the data used in this submission can be accessed. This statement will be typeset if the manuscript is accepted for publication.<br><br>Before publication, authors are required to make all data underlying their findings fully available, without restriction. Review our PLOS Data Policy page for detailed information on this policy. Instructions for writing your Data Availability statement can be accessed via the Instructions link below.	The data used in this paper is available upon request. The code for validation of the citizen scientist collected is available on GitHub is available at the link https://github.com/CiSciUganda/data_validation/blob/main/Automatic_KOBO.

Manuscript
Click here to access/download;Manuscript;Tumusiime et al Manuscript.pdf

Citizens can help to map putative transmission sites for snail-borne diseases

Julius Tumusiime [a,b,1*], *Noelia Valderrama Bhraunxs* [c,d,1], *Grace Kagoro-Rugunda* [a], *Daisy Namirembe* [a], *Christian Albrecht* [b,c], *Ronald Twongyirwe* [e], *Casim Umba Tolo* [a], *Liesbet Jacobs* [d,e] & *Tine Huyse* [c]

[a] Department of Biology, Mbarara University of Science and Technology, Mbarara, Uganda

[b] Institute of Animal Ecology and Systematics, Justus Liebig University Giessen, Giessen, Germany

[c] Department of Biology, Royal Museum for Central Africa, Tervuren, Belgium

[d] Department of Earth and Environmental Sciences, KU Leuven, Leuven, Belgium

[e] Ecosystem and Landscape Dynamics, Institute for Biodiversity and Ecosystem Dynamics, University of Amsterdam, Amsterdam, The Netherlands

[f] Department of Environment and Livelihoods Support Systems, Mbarara University of Science and Technology, Mbarara, Uganda

* To whom correspondence may be addressed. Email: jtumusiime90@must.ac.ug

[1] Equal contribution

Abstract

Introduction

Schistosomiasis is a snail-borne disease that affects over 200 million people globally, 90% living in Sub-Saharan Africa. Similarly, fasciolosis - an emerging zoonotic snail-borne disease, causes significant damage to human health and the livestock industry. However, shortage of snail experts and data hinders implementing the World Health Organisation recommendations on snail control to supplement drug treatment towards disease elimination. Therefore, we adopted a citizen science approach to snail monitoring of *Biomphalaria*, *Bulinus* and *Radix* intermediate hosts of snail-borne diseases.

Materials and methods

Snail data was collected weekly by 25 trained citizen scientists (CSs) at 76 sites around southern Lake Albert (Uganda) for 20 months. At each site, snails were searched for 30 minutes, sorted, target snail hosts identified to genus level, counted and data submitted through a smartphone application. The quality of this data was assessed by comparing it to monthly data collected by an 'expert' malacologist using the same sampling protocol. Generalised binomial logistic and linear mixed-effects models were used to analyse the variables for agreement between the CSs and expert.

Findings

The binary agreement in presence/absence of *Biomphalaria*, *Bulinus* and *Radix* snails reported by the expert and CSs ranged between 70% and 86% (900 reports) with an average of 17% false negatives (sites wrongly defined as snail-free). The agreement for *Biomphalaria* and *Radix* increased with snail abundance, while false negatives decreased when the number of snails collected by citizens were aggregated per month. Site type significantly predicted binary agreement, which was lowest at lake sites (55%) and highest at spring sites (99%) with variations across genera. Similar temporal trends in snail

abundance were recorded despite the expert reporting higher abundance. However, the relative abundance was consistent across site types. The match between the sites with highest *Biomphalaria* spp. abundance identified by CSs and expert was consistently high (~84.1%) and increased over time.

Conclusions and recommendations

Our results demonstrate the potential of citizen science to map putative schistosomiasis transmission sites. We therefore argue that this inclusive, powerful and cost-effective approach can be more sustainable than top-down monitoring and intervention campaigns.

Author summary

Schistosomiasis is a snail-borne disease of great public health importance. Since drug treatment does not suffice to control the disease, the World Health Organisation now recommends including snail control and community involvement. Here we demonstrate how local inhabitants of an endemic region, after training, can detect snail populations with acceptable accuracy, at significantly lower cost. This citizen science approach could therefore generate unprecedented datasets in terms of spatiotemporal resolution and coverage. Moreover, it empowers communities and increases knowledge on snail-borne diseases and their control and prevention. We therefore argue that this community-based approach presents a valuable and sustainable compliment to classical surveillance programs, especially in remote areas, thereby generating the much-anticipated data and community support for targeted snail control.

Keywords: Citizen science; data validation; inter-rater reliability; schistosomiasis; fasciolosis; snail control

Introduction

Snail-borne parasitic diseases form a major public and veterinary health burden, especially in Sub-Saharan Africa (SSA). The most prevalent diseases are schistosomiasis and fasciolosis, caused by *Schistosoma* and *Fasciola* worms, respectively. These parasites rely on freshwater snails of the genera *Biomphalaria* and *Bulinus* (for *Schistosoma*), and *Radix* (for *Fasciola*) to complete their life cycle [1–3]. In its chronic form, schistosomiasis can lead to severe health issues such as hepatic fibrosis, bladder cancer, infertility and/or stunted growth, and reduced learning ability in children [4]. Schistosomiasis has a global burden of approximately 1.43 million disability-adjusted life years [5]. In Africa, fasciolosis (liver fluke disease) predominantly affects livestock and it has been estimated to cause annual losses worth billions of dollars. For instance in Uganda, bovine fasciolosis prevalence in Kampala abattoirs is as high as 84% causing an estimated annual loss of US$ 92.4 million due to condemned liver alone [6]. Currently, the primary approach to controlling schistosomiasis is mass drug administration (MDA) among school-aged children [7]. Despite a recent modelling study using survey data from 44 countries in SSA indicating a considerable decrease in overall prevalence in the last decades [7,8], schistosomiasis continues to (re-)emerge and experts agree that MDA alone is not sufficient [9–12]. Sokolow and colleagues evaluated control programs in 83 countries/territories and estimated that interventions for schistosomiasis that integrate snail control result in a 92% reduction in prevalence, compared to a 37% reduction in programs with no or limited snail control [13]. Simulation studies also showed an additional reduction in total disability of 40% when snail control is added to MDA [12]. In light of these findings, the World Health Organization (WHO) now advises complementing drug treatment with snail control and community involvement in the WHO roadmap for schistosomiasis elimination by 2030 [14]. Additionally, snail control will also impact animal diseases such as bovine schistosomiasis, amphistomiasis and fasciolosis, that are transmitted by the same or sympatric snail species, aligning with the One Health approach and steering away from a single-disease focus.

To minimize the environmental impact of snail control, a focal or targeted approach is necessary, whether it involves physical, chemical or biological methods [9,10,15]. However, to design effective snail control interventions, we need detailed information on snail population dynamics at fine spatial and temporal scales, and a thorough understanding of the biotic and abiotic factors that influence these dynamics. Unfortunately, there is currently a severe shortage of trained malacologists worldwide, particularly in SSA, where schistosomiasis is most prevalent [10,16].

One promising method to scale up data collection is to develop a citizen science-centred approach [17], where members of the general public are involved in scientific efforts within their communities [18,19]. Citizen science involves collecting large volumes of research data, or conducting scientific experiments that are often in response to the local community or societal needs. While citizen science is still in its early stages in Africa [17,20], and never been applied in schistosomiasis control, it has recently been proven to be a viable alternative or complement to monitor vectors of malaria, Lyme disease, yellow fever, and Zika [21–25].

However, the quality and reliability of citizen-generated data is often questioned [26–28]. Investigating data quality in citizen science projects is not straightforward since the nature and amount of incoming data are project-dependent, and so are the required protocols for data quality [27]. Successful projects often deploy a combination of strategies including iterative designs, methods standardization, systematic capture, classification and data-entry procedures, refresher training for citizens, ongoing feedback, registration of metadata, statistical analysis to detect and correct potential bias, and expert validation [27,29]. However, implementing such tailored measures to monitor and evaluate data quality can be limited by associated costs in terms of human and material resources [28,30,31]. Nonetheless, thorough validation processes are necessary to harness the potential of citizen-generated data for advancing scientific knowledge and guiding evidence-based policy [32,33].

In this study, we analyse data obtained in the project 'Action Towards Reducing Aquatic snail-borne Parasitic diseases' (ATRAP), which established a citizen scientist network in Uganda consisting of 25 citizens that monitored snail populations at fixed sites. We compared this dataset with data obtained by an expert malacologist who visited the same sites using the same sampling protocol. Our aim is to assess the extent of agreement in (i) snail occurrence (presence/absence) - referred to as binary agreement, and (ii) snail abundance - evaluated as consistency and numerical agreement. By exploring the extent and type of observational biases in citizen-generated data, we aim to investigate the potential of citizen science in mapping putative transmission sites for guiding targeted snail control.

Materials and Methods

The study was conducted in the southern part of Lake Albert that is characterised by diverse hydrological settings – upland small streams and rivers (here categorised as stream site type), swamps and stationary pools (categorised as wetland), artificial spring wells (categorised as spring), and lacustrine shorelines and sheltered bays (categorised as lake). The sites are frequently used by the local communities increasing the risk of snail-borne disease infections. See *SI Appendix* Fig S1 for a detailed description of sampling site types.

The citizen scientists (hereafter referred to as CSs) were recruited upon recommendation by the community leadership in collaboration with the ATRAP team [34]. The established network has been operational from March 2020 until February 2023 and each CS reported data on a weekly basis at the designated water contact sites (2-4 per CS) in their village. The 25 CSs were trained in snail sampling and identification to genus level, focusing on species belonging to *Biomphalaria, Bulinus* and/or *Radix*. The procedures for site selection, training of CSs, snail sampling, data submission via the Kobo collect mobile application [35], and validation were described in detail by Brees et al. [34].

The reporting protocol consisted of multiple-choice and open questions about the presence, identity, and quantity of snails, GPS location, sampling duration, and time. At each mapped water contact site,

sampling occurred within a diameter of 10 m. The CSs, wearing appropriate protective gear (latex gloves and gumboots), actively searched for snails for ca. 30 minutes using a hand-held scoop net attached to a two-meter-long metallic handle. The collected snails were sorted into groups (genus level) based on their morphological features as learned during the CSs' training. *Biomphalaria*, *Bulinus* and *Radix* snails were counted and recorded in the questionnaire, while snails from other genera were classified as 'pool'. Subsequently, all the snail groups were placed on a graph paper and photographed at close range. The picture was then uploaded to a central server for later verification. All the collected snails were returned to the site after recording the information to minimise environmental impact.

The verification protocol was designed to minimise *reporting errors*, i.e. the difference between sampled data by the CSs and the data submitted in the questionnaire [34]. Reporting errors minimization was done by using skip logic and favouring multiple-choice questions, setting up a robust semi-automatic validation protocol that flags faulty entries, providing regular feedback, and organising an annual refresher training for the CSs. The data validation code developed in Python is available on GitHub at https://github.com/CiSciUganda/data_validation/blob/main/Automatic_KOBO. See Brees et al. [34] for a detailed description of semi-automatic validation.

Next to the automatic verifications, manual verifications were done by an experienced researcher (NVB) based on the submitted pictures. The pictures of the remaining snail species labelled as 'pool' were used to verify any false absence. Any snail misclassification, inaccurate sampling location, and incorrect scoop time (correct scoop time = 30 ± 10 minutes) were identified and flagged as reporting errors. Each of the snail genera (*Biomphalaria*, *Bulinus* and *Radix*) was independently verified since reporting errors for one genus do not affect the validity of correct reports for another genus. All reports with reporting errors were filtered out (<2.5% of total reports) before further analysis.

On the other hand, *observational errors* relate to the difference between the snail presence and abundance reported by the citizens, and the (actual) *in situ* situations. This bias could not be tested by

Brees et al. [34] due to a lack of ‘ground-truth’ data. Therefore, a trained malacologist (JT) (hereafter referred to as the ‘expert’) visited the same sites that were monitored by the CSs weekly on a monthly basis, following the same standardised snail collection protocol. The ‘expert’ dataset was used as a proxy for ~~the~~ ‘ground truth’, allowing a thorough comparison with the CSs data. Each CS sampled two to four water contact sites, but three of these sites were excluded from our analysis as the expert was unable to regularly access these remote sites. As a result, 73 out of 76 sites monitored by 24 of 25 CSs on a weekly basis were considered for data analysis. We included data from June 2020 to April 2022, when the expert stopped the monthly sampling campaigns. Due to the flooding of Lake Albert at the end of 2019 through 2021, the initially selected sampling locations (14 lake sites) shifted in space. Therefore, the CSs and expert agreed on new locations to monitor each time the water levels changed.

The expert identified all snails, including the ‘pool’ to species level using morphological identification described by Brown [36] and Mandahl-Barth [37]. The snails collected by the expert were kept for shedding experiments and for molecular analysis to identify snail infection, and for species identification. However, in the last four months of snail sampling, all snails were returned to the site after enumeration to assess the bias potentially introduced by snail removal on agreement/disagreement between the raters. The slight deviation of the expert from the CS protocol and the possible consequences for data comparison are discussed in *SI Appendix* Fig S2 and Fig S3 showing no major source of divergence.

Statistical analysis

Snail presence/absence data

The data points collected by the CSs and the expert were matched in space and time, considering the closest observation within a period of seven days. The closest time points were selected for the pairwise comparison to minimise natural differences due to mortality, birth, and passive or active dispersal of snails between both time points [38].

Subsequently, the reported snail abundance was simplified to occupancy records (presence/absence) and compared in terms of binary agreement or disagreement. The extent of agreement between the CSs and expert data was expressed as a percentage of all paired data across the site types. Site type was used as the grouping variable as the categories (lake, spring, stream or wetland) have distinctive characteristics. Agreement/disagreement was split into four components of the confusion matrix. Agreement consisted of true positives (TP) when both the expert and CSs independently recorded snail presence at a site and true negatives (TN) when both reported snail absence at a site. Disagreement was split into false negatives (FN) when the expert recorded snail presence at a site while the CS did not, and false positives (FP) when the CS recorded snail presence at a site, but the expert did not. False positives and false negatives in this case are thus considered relative to the expert data (the so-called ground truth). As such, 'false' observations in our case can thus either be caused by observation errors by CSs, the expert, or actual differences in occurrence due to changes in the local conditions (paired samples can be as much as 7 days apart).

Sensitivity describes how well an observer detects the presence of objects/phenomena when they are actually present [39]. It is calculated by dividing the TP by the sum of TP and FN and expressed as a percentage. Expert-collected snail data was used as the reference for the computation of sensitivity of CSs in detecting snail presence at a site. A statistical analysis was then performed to further investigate why the expert and CS data might differ. The variables considered for this are summarised in *SI Appendix* Table S1. A generalised binomial logistic mixed-effects regression (GLMER) was used to analyse the binary agreement of the expert with the CS snail presence/absence data. GLMER treats data points as grouped (hierarchical) considering the nested nature of data based on a random effect such as repeated measurements of the same subject [40]. Our dataset consisted of repeated observations of sampling locations that are nested at the CS level (*SI Appendix* Fig S4). Site type, CS ID, the difference in sampling dates between CS and expert, and snail abundance were tested as predictors (fixed effects) of binary

agreement between the expert and CS snail presence/absence data. Sampling location (site) nested between CS ID was considered as a random effect and included as a random intercept to avoid autocorrelation (repeated observations at the same site and/or by the same CS). The snail abundance reported by the expert was transformed to a count per 30 minutes and used as an independent predictor of binary agreement in presence/absence of snails at a site.

A generalised linear mixed model framework of the GLMER is described following Ugwu and Zewotir [41]. Here Y_{ij} is the binary response (agree/disagree) in the presence of snails between the CS at the *j*th location and the expert, with $P(Y_{ij} = 1)$ denoting the probability that at an *i*th time and the *j*th site, the CS agrees in snail occupancy with the expert. The GLMER then takes the following form:

$$P(Y_{ij} = 1) = \frac{exp\ (\eta_{ij})}{1 + exp\ (\eta_{ij})} \quad \text{Eq. 1}$$

With

$$\eta_{ij} = \beta_0 + \beta_1.ID_j + \beta_2.ST_j + \beta_3.DD_{ij} + \beta_4.NS_{ij} + \gamma \ .(1|ID_j/Site_j) \quad \text{Eq. 2}$$

Where, ST_j is the site type, DD_{ij} is the sampling date difference, NS_{ij} is number of snails for each genus, ID_j is the citizen scientist identification number at $Site_j$ and $Site_j$ is the sampling location. β denotes the fixed effects coefficients while γ denotes random effect coefficients.

A simplified model for each of the genera was developed by a manual stepwise step-up forward inclusion procedure. The inclusion of independent variables was based on a minimization of the Akaike information criterion (AIC) to balance model complexity and explanatory power [40]. The model was explored for collinearity through the calculation of the generalised variance inflation factor (VIF) values. The VIF of three was considered the threshold for collinearity [42]. The reduction in model deviance

(chi-square) was used to evaluate improvement in model fit after inclusion of independent variables compared to the null model. The function *somers2()* was used to compute Somers' D_{xy} and the C index of concordance [43]. Somers' D_{xy} is a rank correlation between predicted probabilities and observed responses and takes values ranging from 0 (randomness) and 1 (perfect prediction), with values greater than 0.5 considered meaningful. On the other hand, C is an index of concordance between the predicted probability and observed response. Values of C of 0.5 indicate randomness, 0.8 indicate the model has real predictive capacity while one (1) indicates perfect prediction [43]. All the analyses were done in R statistical software (v4.1.0; R Core Team 2021).

Snail abundance data

An exploratory analysis was performed to visualize the concordance between the snail abundance reported by the citizens and the one reported by the expert through time; as well as relative to the site types. After visualisation, the quantitative similarity- *raters' reliability*- between reported snail abundance was measured. For this analysis, only paired observations (previously adjusted to a scoop time of 30 minutes) where both the CSs and the expert reported snail presence (i.e. the TP pairs) were considered. We assess how well snail abundance is reflected by the CSs when both expert and CS detect the respective snail genus. For a detailed measure of these similarities, the data were analysed in subsets considering the site type, and the concept of reliability subdivided into consistency and numerical agreement.

Consistency is here defined as the resemblance between rankings of abundance by both rater types (CS and the expert). To compare ordinal association between two ranks, Kendall's Tau-b correlation coefficient was used [44,45]. Kendall's Tau-b allows assessing how both rater types agree in the determination of abundance ranking of the sites. Particularly, Kendall's Tau-b coefficient is used when the analysed data presents ties within a group (observations when a certain group has two or more

instances with the same quantity of snails in their sampling). Kendall's Tau-b coefficient was calculated from the following formula [44,45]:

$$\hat{t}_b = \frac{C - D}{\sqrt{\left[\frac{n(n-1)}{2} - T_x\right]\left[\frac{n(n-1)}{2} - T_y\right]}}$$

Eq. 3

$$T_x = \sum_{p=1}^{ex} \frac{n_p\left(n_p - 1\right)}{2} \qquad T_y = \sum_{q=1}^{cs} \frac{n_q\left(n_q - 1\right)}{2}$$

Eq. 4

where C represents the concordant pairs, defined as those where the sign of (X_p – X_q) equals the sign of (Y_p-Y_q) whereby X_p and X_q are ranks of two observations by the expert and Y_p and Y_q are the paired observations made by the CSs and D the discordant ones where the sign of (X_p – X_q) is opposite to the sign of (Y_p-Y_q) [45]; n represents the number of paired observations, T_x and T_y represent the number of tied observations as defined above for either the CS data or the expert data, respectively; and p, q represents observations of snail abundance at sites where expert and CS sampled within less than one week of each other. Kendall's Tau-b coefficient range from -1 to 1, with 0 indicating no correlation and (+ or -) 1 indicating a perfect correlation. For this analysis, each TP paired observation was used to assess the overall agreement in rank, without considering the nested structure of the data.

In contrast to the binary agreement defined above, the numerical agreement was defined in terms of the algebraic differences between the values reported by the raters relative to an absolute zero point; and calculated through Krippendorff's alpha coefficient. Krippendorff's alpha measures numerical agreement while correcting for chance agreement and is calculated from the following formula [46]:

$$\alpha = 1 - \frac{D_o}{D_e}$$

Eq. 5

whereby D_o represents the observed numerical disagreement and D_e represents the numerical disagreement that is attributable to chance. Both measures rely on values specific to frequencies reported in coincidence matrices for which the construction is specific to the data type, which in our case is ratio metric differences. For a full description of the computation of α, we refer to Krippendorff [46]. Algebraically, when observers have a perfect match (no disagreement) α is equal to 1; but Krippendorff's α coefficient usually ranges from -1 to 1, with -1 being opposite numerical agreement, 0 no numerical agreement or numerical agreement due to chance and 1 perfect numerical agreement [46].

Additionally, we analysed the factors that could influence differences between the snail abundance reported by CSs and the expert. For this purpose, a linear mixed-effect model (GLMMs) was computed considering a nested structure due to the multiple observations from the same locations made by each CS (*SI Appendix* Fig S4). We used linear mixed-effects regression to explain the difference of the snail abundance reported between CSs and expert as follows:

$$\Delta A_{ij} = \beta_0 + \beta_1 . DD_{ij} + \beta_2 . ST_j + \beta_3 . date_{ij} + \gamma_2 . (1|ID_j/site_j)$$

Eq.6

The counting difference between reported snail abundance (TP: true positives, cases when the CSs and the expert reported snails) for observation *i* per location j, ΔA_{ij}, has a normal distribution with mean μ_{ij} which is modeled as a linear function of the predictors (DD_{ij}: time difference in days between sampling made by the expert and CSs; ST_j: site type; and $date_{ij}$: scaled date of sampling) as well as a random intercept term for each ID_j: CS ID and $site_j$: sampling location. The scaled data represents the number of days since the first day of sampling. The collinearity of the predictors was evaluated through VIF values with a threshold of three [42]. The data of each snail genus was analysed per group: (a) data whereby snail abundance reported by expert is larger than snail abundance reported by CSs, and (b) data

with lower abundance reported by the expert compared to the CSs, as we aimed to explore which factors could underly both situations.

Results

The expert collected a total of 1,382 data reports, while the CSs collected 4,034 reports. After filtering for differences in sampling dates, errors in snail identification, and sampling time, the datasets from the expert malacologist and CSs were merged. This resulted in a total of 907 paired reports on *Biomphalaria* spp., 892 on *Bulinus* spp. and 898 on *Radix* sp. were obtained. SI *Appendix* Fig S5 illustrates the total abundance of the three snail genera collected by the expert over a 20-month period. It is worth noting that out of the 73 sites observed, 72 sites (98.6%) contained at least one of the three snail genera during the observed period.

Presence and absence

Biomphalaria snails were the most abundant and widely distributed among the three genera, being recorded at 68 (93%) sites (see *SI Appendix* Fig S5). In approximately 76% of the paired reports on *Biomphalaria*, there was a binary agreement in snail occupancy at a site (presence/absence) between the expert and CSs. However, the frequency of agreement varied across different site types, as depicted in Fig1A. The highest agreement was observed at wetland sites (86%), followed by streams (78%), and spring wells (72%), while lake sites had the lowest agreement (60%; N = 907). Overall, the true positive rate (sensitivity) was determined to be 74.6%.

Bulinus snails exhibit lower abundance and a patchy distribution within the study area. Approximately 86% of the paired reports on *Bulinus* showed binary agreement between the CSs and the expert. However, the sensitivity of *Bulinus* snail detection by the CSs was found to be low (43.4%; Fig 1B).

Conversely, the *Radix* group displayed the lowest binary agreement (70%) but with a moderate sensitivity (59.5%; see Fig 1C).

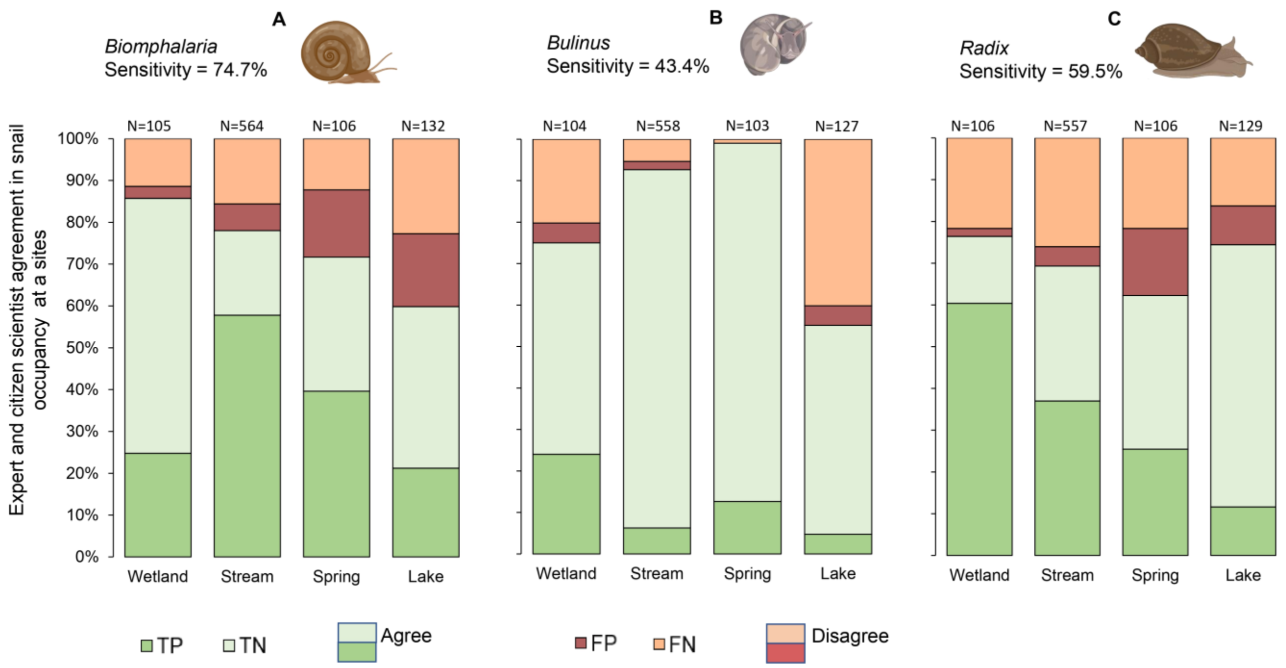

Fig 1. Agreement between the expert and citizen scientists collected data on the presence/absence of *Biomphalaria* (A), *Bulinus* (B) and *Radix* (C) snails at different site types.

The GLMER model for *Biomphalaria* was defined as $P(Y = 1) \sim SN_{i,j} + ST_j + DD_{i,j} + 1 \mid ID_j/Site_j$ (refer to Fig 2A-C), with a probability of binary agreement, P(Y = 1), equal to 0.76. The fitted model indicated a significant prediction of agreement by the independent variables (C = 0.795, Somers' D_{xy} = 0.590; N = 907), as evidenced by the reduction in deviance compared to the random effects model (chi-square (5) = 35.5; $p < 0.01$).

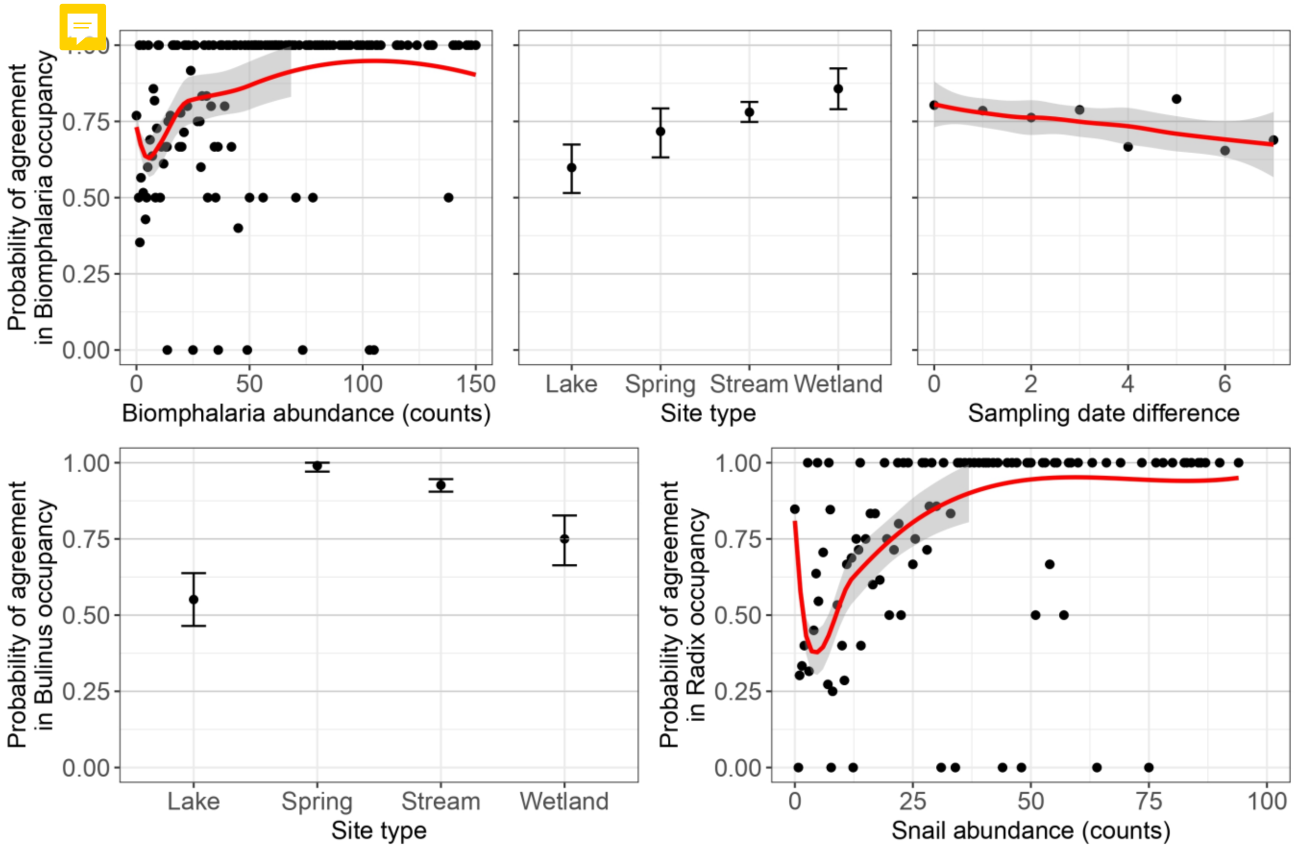

Fig 2. Variables influencing observed mean probabilities of agreement in snail presence/absence: *Biomphalaria* spp. presence/absence by snail abundance (A), site type (B) and sampling date difference (C); in *Bulinus* spp. presence/absence by site type (D); in *Radix* presence/absence by snail abundance (E). The 95% confidence level is indicated by the grey limits on the fitted red line for continuous data and by the error bars for site type.

In general, the likelihood of binary agreement showed a significant increase as the abundance of *Biomphalaria* snails increased (odds ratio [OR] = 1.02; $p < 0.001$), particularly in wetland sites (OR = 4.30; $p = 0.002$) or stream sites (OR = 2.33, p = 0.008) (see Fig 2A and B). However, the binary agreement significantly decreased with an increase in the difference in sampling dates between the expert and the CSs (OR = 0.91; $p = 0.02$; Fig 2C), indicating that disagreement is at least partially driven by differences in sampling date, rather than errors committed by the sampler(s).

The order in which the expert and CSs conducted the sampling (whether the expert sampled before, after, or on the same day as the CS) was not a significant predictor of binary agreement for all three genera ($p > 0.05$), suggesting that the expert's snail removal had minimal impact on snail presence/absence (*SI Appendix* Fig S2 and S3). The reduction in model deviance (chi-square) when including sampling order was small, ranging from 2.1 to 2.7 and statistically insignificant. Furthermore, the effect of snail replacement by the expert in the last four months of snail sampling on the binary agreement between CS and expert data could not conclusively be determined. The agreement decreased for *Biomphalaria*, remained the same for *Bulinus* and increased for *Radix* (*SI Appendix* Fig S2 and S3). Detailed information on the predictors of agreement in the presence/absence of *Biomphalaria* spp., including effect sizes, confidence intervals (CIs) and ORs, can be found in *SI Appendix* Table S2.

When fitting a GLMER model for agreement in *Bulinus* spp. presence/absence, we found that only site type significantly explained the agreement (Fig 2D), with a probability, P(Y = 1), equal to 0.86. Spring sites (OR = 104.7; p <0.001), stream sites (OR = 11.8; p <0.001) and wetland sites (OR = 3.4; p = 0.032) exhibit a higher likelihood of achieving binary agreement compared to lake sites (Fig 2D). The fitted model demonstrated a significant improvement over the reference random effects model when site type was included as a fixed variable (chi-square (3) = 42.3; $p < 0.01$; C = 0.889; $D_{xy} = 0.778$; N = 892). For *Radix*, the agreement in presence/absence was determined by the formula: $P(Y=1) \sim SN_{i,j} + 1 \mid ID_j/Site_j$, with a probability of binary agreement, P(Y=1), equal to 0.70. The probability of agreement between the CSs and the expert increased significantly with an increase in *Radix* sp. abundance (Fig 2E; OR = 1.01; $p = 0.031$). Despite the simplicity of the model with only one fixed variable, the explanatory power of snail abundance was relatively high (C = 0.780; $D_{xy} = 0.560$; N = 867). Other factors such as site type and the difference in sampling dates between the expert and the CSs did not significantly explain the binary agreement in detecting *Radix* snail presence/absence at a site.

Snail Abundance

The total number of true positives cases (TP) were 422 for *Biomphalaria* spp., 79 for *Bulinus* spp., and 313 for *Radix*. A visual analysis of the data (Fig 3A) reveals that *Biomphalaria* snails exhibited the highest accumulated abundance, while *Bulinus* snails had the lowest abundance. Furthermore, the expert consistently reported tended higher abundance compared to the CSs for all snail genera. However, there appears to be a general agreement between the CSs and the expert regarding temporal trends, with minor variations observed (Fig 3A). Additionally, when comparing the snail abundance data between CSs and the expert, the relative dominance of each genus by site type remained consistent (Fig 3B).

To visually assess the agreement in rankings across time and space, Fig 3C illustrates the overlap in the top 15 sites (ranked by abundance) for *Biomphalaria* spp. in all comparisons of expert and CS data (TP, TN, FP, FN). All months but one exhibit more than 75% of concordance, with an average concordance of 84.1%. Additionally, the refresher training conducted in January 2021 (Fig 3D) resulted in significantly higher agreement in the subsequent months ($t = -2.59$; $p = 0.023$; $df = 12.739$).

Regarding the assessment of raters' reliability (Table 1), the consistency results (Kendall's Tau-b) displayed a constantly significant correlation coefficient for stream and wetland sites across all three genera. Among the snail genera, *Bulinus* presented the highest values of Kendall's Tau-b, implying strong relationships between the ranking of its abundance reported by the expert and the CSs at each site type, particularly at lake and stream sites. However, it should be noted that there were limited TP observations around the lake (n=6). *Biomphalaria* and *Radix* snail abundance had the highest values of consistency among raters at wetland sites (0.541 and 0.381, respectively). For *Bulinus*, the lake site type had the highest Kendall's Tau-b but was not considered due to the small sample size (n=6). The second-highest consistency for *Bulinus* corresponded to the stream site type where snails from this genus are more abundant (0.637).

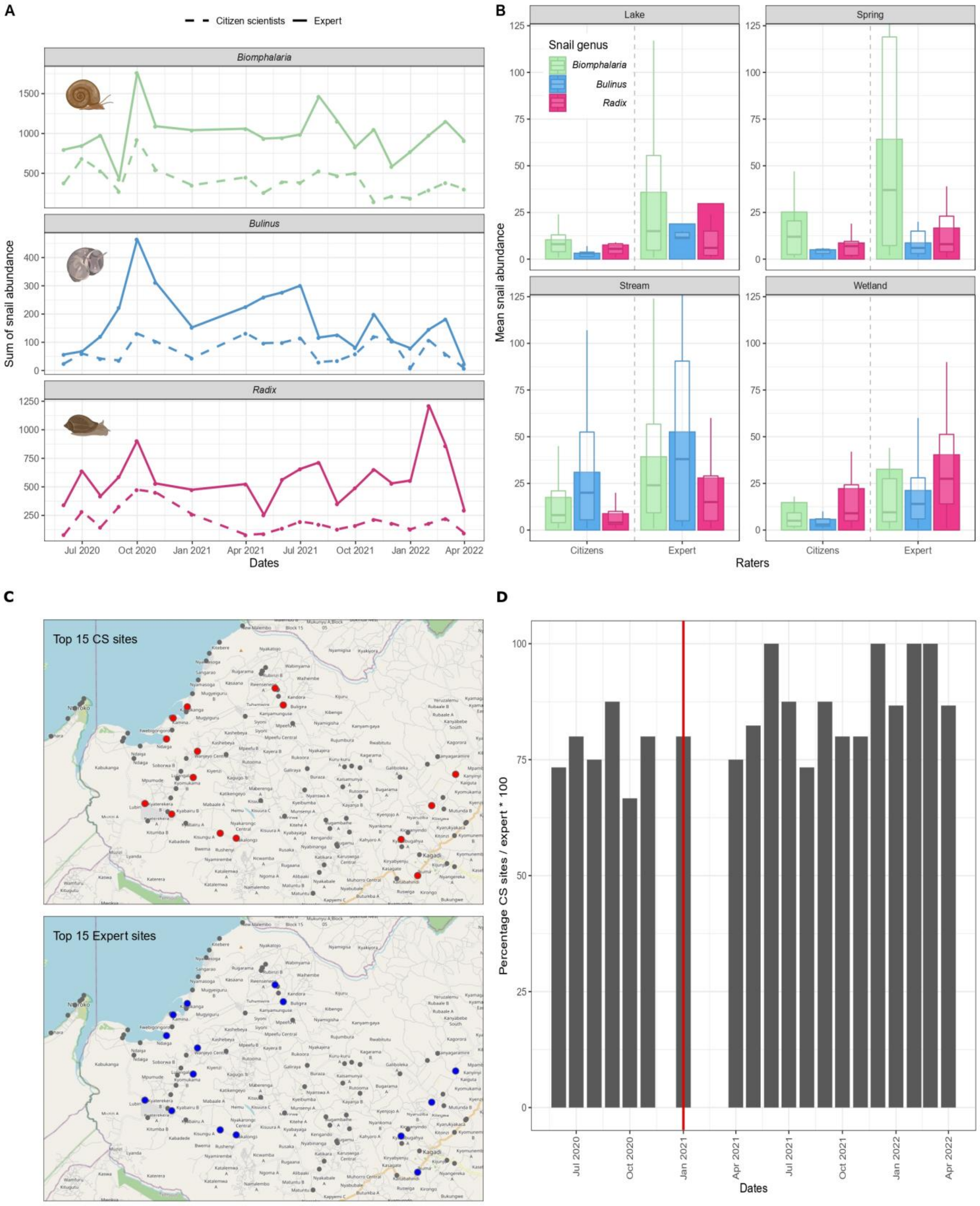

Fig 3. Spatial and temporal trends comparison of snail abundance reported by citizen scientists and expert. Sum of snail abundance over time per type of rater (A). Mean snail abundance per site type and

type of rater (limit of y-axis has been set to 120) (B). For those instances where paired reports are available for more than 40 sites, we consider the agreement in the top 15 locations in terms of abundance. Top 15 locations with the highest abundance of *Biomphalaria* spp. in February 2022 (citizen reports on the top, expert on the bottom, results from the other months showed similar trends) (C). Percentage of concordance between top 15 sites of *Biomphalaria* spp. abundance reported by raters over time, the red line indicates the refresher.

In terms of numerical agreement (Krippendorff's α), positive coefficients were observed for spring, stream and wetland site types, indicating a positive relationship between the snail abundance reported by CSs and the expert. The stream site type exhibited the highest numerical agreement for *Bulinus* spp. (0.612), while the wetland site type showed the highest numerical agreement for *Biomphalaria* spp. (0.457). Although the values of the numerical agreement are lower than the ones of consistency, this outcome was expected since the numerical agreement metric is stricter than the one of consistency.

Table 1: Raters' reliability assessment: consistency and agreement of snail abundance reported by citizens scientists and expert

Site	*Biomphalaria* spp.				*Bulinus* spp.				*Radix* sp.			
		Cons		Agree		Cons		Agree		Cons		Agree
	N	K. Tau-b	*p*	K. α	N	K. Tau-b	*p*	K. α	N	K. Tau-b	*p*	K. α
Lake	28	-0.15	0.27	-0.10	6	0.74	0.05	-0.12	16	0.23	0.28	0.18
Spring	42	0.41	<.01	0.27	13	0.34	0.12	0.27	27	0.11	0.45	0.06
Stream	326	0.38	<.01	0.19	35	0.64	<.01	0.61	206	0.30	<.01	0.08
Wetland	26	0.54	<.01	0.46	25	0.49	<.01	0.06	64	0.38	<.01	0.25

N is the sample size; Cons is consistency; K. Tau-b is Kendall's Tau-b; Agree is agreement and K. α is Krippendorff's alpha. Grey cells were discarded due to a small sample size.

In our analysis, we examined the factors influencing the disparity between snail abundance reported by the expert and the CSs, and we did not identify any variables that reached statistical significance ($p<0.05$). For more detailed information about this, please refer to *SI Appendix* Table S3-S4.

Discussion

This study aimed to assess the suitability of a citizen science approach for monitoring snail intermediate hosts, with the goal of guiding targeted snail control efforts and to support species distribution modelling. In order to evaluate the quality of the collected data in relation to these objectives, we employed the concept of 'fitness for use' as suggested by Parrish et al. [21]. To ensure the generation of relevant and high-quality data, we purposively selected participants and developed a rigorous sampling protocol with fixed sampling times and sites [34]. This deliberate approach helps to mitigate issues related to sampling bias, which are commonly encountered in many citizen science projects that rely on opportunistic participation and self-selection by volunteers, as seen in projects like *Mosquito alert* and *Spider in da house* [21,47]. However, in order to assess the reliability of citizen-collected data, we chose to compare it with temporally matched data collected by an expert at the same locations, considering the expert data as the 'ground truth'. This choice, and the snail sampling method (see below), has some repercussions that we need to discuss before going into the results. 'Ground truth' is difficult to establish because freshwater snail species are difficult to sample exhaustively for several reasons. Firstly, *Biomphalaria*, *Bulinus* and *Radix* snails are relatively small, with juvenile stages measuring only a few millimetres, usually hidden in the vegetation or sediment [48]. Secondly, their dispersal is not spatially continuous but rather stratified, involving a combination of passive long-range dispersal through water currents, waterbirds, and humans, as well as active short-range movements in search of food, mates or shelter from predators, heat and strong currents [49]. Additionally, circadian vertical migration has also been observed [50]. Consequently, observational inconsistencies are likely to occur, even when the same

expert samples the same site at different times of the day. It is therefore worth noting that our quality control measures might be overly stringent, as some of the observation errors could be attributed to factors unrelated to the CSs' performance.

In the next paragraphs we discuss two fundamental questions for prospects involving citizen scientists in snail monitoring; 1) can CSs determine snail presence at a site? And if so, 2) can they reliably monitor snail abundance?

Can citizens correctly monitor snail presence and absence?

Our study demonstrates a substantial level of agreement between CS-collected and expert-collected data regarding the presence or absence of snails at a given site. The binary agreement rates were highest for *Bulinus* (86%), followed by *Biomphalaria* (76%) and *Radix* (70%). These agreement values are relatively high compared to the 50-80% reported among marine divers as CSs compared to experts in recording the presence of biological taxa and litter [51], but similar to the 79% reported in the *Mosquito alert* project [21]. However, our binary agreement was influenced by factors such as snail abundance and site type. For example, presence/absence of *Biomphalaria* spp. was more likely to match between the expert and CSs at sites with higher snail abundance (see Fig 2A) such as wetland sites (86%), while it was lowest at lake sites (60%). These findings could be explained by the fact that snail population dynamics are influenced by habitat type and hydrological conditions [52]. Wetland sites in the study area are relatively stable systems with minimal chances of snail migration due to their shallow lentic nature, small size, and clearly delineated boundaries. Therefore, the chances that two independent observers will find snails at such a site are higher compared to a larger and deeper lake system. Wave action in lakes can also alter the shoreline vegetation and thus snail communities within a few hours in response to heavy winds [53]. Disagreement was also relatively high in spring wells (see Fig 2B), which are regularly unblocked by removing mud and debris, which are substrates that support snails. Spring wells are also usually located in valleys where they are prone to runoffs from heavy rains. The probability

of agreement for *Biomphalaria* occupancy decreased when the difference in the number of days between sampling by the expert and CS increased (Fig 2C). This could be explained by an increasing chance for changing weather events with time, affecting the sampling sites as described above. Previous studies have shown how heavy rains or wave action can lead to passive snail dispersal [54] and subsequently low recapture values as low as 20% after one week [48]. As none of the interaction effects for site type and sampling date difference was significant, none of the site types seem to attenuate the effect of short-term fluctuations significantly, thus corroborating our statement earlier that using expert data as ground truth might be too strict.

Bulinus spp. presence/absence data had the highest binary agreement between the expert and CS data. However, the detection sensitivity was the lowest (43.4%) of the three snail genera studied. Lower values of sensitivity and higher values of specificity are likely when the frequency of occurrence of the event is low [39,55,56]. In our study area, *Bulinus* spp. snails were rarely encountered and when present, in relatively low numbers. For most of the sampling months (15/20), the monthly total abundance of *Bulinus* reported by all raters was less than 250 snails across the 73 sites (see Fig 3A). Consequently, the class with true negatives (TN) was very high (77%). The CSs were, therefore, able to correctly identify sites where *Bulinus* snails did not occur, increasing the chances of binary agreement with the expert (86%). Similar trends were found in *Mosquito alert* for the comparison of mosquito trapping by experts and mosquito reporting by CSs in areas with low mosquito abundance [21].

Radix snail presence/absence agreement between the CSs and the expert was the lowest of the three genera and the agreement was significantly predicted by snail abundance. *Radix* snails tend to attach firmly to the substrate and are often found on substrate suspended in fast-flowing waters, unlike *Bulinus* and *Biomphalaria* snails. The large shell aperture relative to the size of the snails [57,58] could provide a large surface area for the foot to attach to the substrate firmly. We observed that sampling *Radix* snails from the water using a scoop net requires extra effort in agitating the substrate, making it more difficult

compared to sampling *Biomphalaria* and *Bulinus*. This could explain why there were more false negatives for *Radix* paired reports (23.6%, N = 898). However, the degree of binary agreement in *Radix* presence/absence increased from 70% to over 80% from September 2021 to February 2022 (see *SI Appendix* Fig S6), corresponding to a period of higher *Radix* abundance reported by the expert as shown in figure 3A. Generally, as the abundance of *Radix* snails increased, the CSs were more likely to agree with the expert, similar to *Biomphalaria* snail data. However, the decline in snail abundance from the peak in February to April 2022 was contrasted by increasing binary agreement in the same period (*SI Appendix* Fig S6). We attribute this to the hands-on refresher training organised in January 2021, which emphasised the snail sampling technique. This underpins the importance of training and feedback in improving CSs' performance [34], especially when high quality data is desired, and when sophisticated sampling procedures and fine-scale morphological identification of organisms are involved [59–63].

The effect of CS ID on presence/absence could not be conclusively analysed due to an increase in model complexity. There is however an apparent association between CS ID and binary agreement in the presence/absence of snails. This association could be reflected by the majority of them performing well (over 50% agreement), and a few performing excellently (>80% agreement), or poorly (<50% agreement) (see *SI Appendix* Fig S7). Future research should investigate the factors behind this inter-individual variation, by studying the link between motivation and data quality for example.

The agreement in presence/absence is more straightforward when both the CS and the expert do (not) find snails at a site (true positives or negatives). However, the consequences of and reasons behind disagreement (false positives or negatives) are more complex. High numbers of false negatives would result in CSs underestimating the risk of schistosomiasis infection at a site. In this study, this risk is partly offset by having frequent monitoring (weekly sampling by CSs). By aggregating weekly reports by CSs and calculating the cumulative number of snails collected per month, the number of false negatives significantly decreased (chi-square (1) = 33.9; $p < 0.01$) for all genera (SI Appendix Fig S8,

Table S5). False positives on the other hand, represent cases where snail pictures uploaded by the CSs are carefully validated for correct snail identification, so the reported snail was actually present. This again illustrates the limitation of our 'ground truth', as discussed above.

Can citizens reliably monitor snail abundance in given site/habitat types?

For all snail genera, snail abundance reported by the CSs were positively correlated with those obtained by the expert. However, the correlation values were lower than those reported in other studies about intercoder reliability [64–66]. Nevertheless, most of these studies focus on medical diagnosis, which involves fewer categories of classification, and therefore fewer opportunities for disagreement. In our study, we used Kendall's Tau-b coefficient to assess the level of consistency for a discrete variable (snail counts), with values from 0 to 282 (see *SI Appendix* Table S1). The high number of reports included in our study (422, 79 and 313 for *Biomphalaria*, *Bulinus* and *Radix* snails, respectively) can significantly impact the estimated marginal distribution of the snail counts. This, in turn, leads to an increased number of possible ranks, resulting in a greater occurrence of discordant pairs, as discussed by Agresti [45]. Consequently, these factors could possibly contribute to lower Kendall-tau values.

Similarly, the results of numerical agreement, as indicated by Krippendorff's alpha, revealed a lower agreement between CSs and the expert compared to other studies evaluating intercoder reliability in a citizen science framework [67–70]. Again, the majority of these studies were focused on the agreement between a limited number of categories (from 2 to 6) whereas we worked with ratio data that considered the algebraic differences between the values reported by the raters. Although using abundance intervals could potentially yield higher alpha values, we opted to retain the highest level of detail in our data. Nonetheless, Berland et al. [69] reported higher values of alpha (>0.8) when assessing intercoder agreement among novices, intermediate analysts and expert analyst counting trees. In our study, the lower values of snail abundance agreement are most likely also partly attributed to the nature of our study subjects, freshwater snails, which, unlike trees, are not immobile (see discussion above).

In line with the reported results of the presence/absence analyses, snails from the genera *Biomphalaria* and *Radix* presented higher values of consistency and numerical agreement in wetlands (Table 1), probably due to the shallow and lentic nature of these water bodies. In contrast, the highest agreement for *Bulinus* snail abundance was observed in stream sites (Table 1), which may be attributed to specific locations where *Bulinus* snails were found, such as Kolanya and Nyamasoga. These sites are characterized by small, slow-flowing streams with confined access points, which support relatively stable populations of *Bulinus*. It is important to note that both Krippendorff's α and Kendall Tau-b are calculated with all paired observations for each genus, and thus the resulting values do not account for the temporal structure of the data. When calculating these metrics for each timestep, we observe less variations (especially for *Bulinus* snails) in Krippendorff's α than in Kendall Tau-b which is more likely to be affected by sample size (see SI *Appendix* Fig S9).

A plausible explanation for the differences in snail abundance between CSs and the expert is the task difficulty. Determining the presence or absence of a particular snail genus requires relatively low skill, which CSs would already acquire after the initial malacological training. However, obtaining reliable abundance data comparable to the ground-truth data (expert data) requires that, in addition to identifying the potential microhabitats of the snails at the sampling sites, a vigorous and continuous sampling for 30 minutes should follow, which demands considerable physical effort and dedication. In the latter, individual factors such as motivation or skill, could contribute to the observed variations in performance among CSs (see *SI Appendix* Fig S7). The observed increase in agreement in the ranking of high abundance sites over time supports the hypothesis of task difficulty, indicating that CSs improve their performance with more practice and experience, but also after the refresher training that stressed the importance of correct sampling (Fig 3D).

For all evaluated snail genera, consistency values were higher than numerical agreement, suggesting that using proportional abundance (Fig 3B) can be a more effective approach than exact numerical agreement, although both metrics remain relatively low and are strongly influenced by the site type.

Can citizens help to map putative schistosomiasis transmission sites to guide targeted snail control?

Our study provides evidence of high agreement in presence/absence and spatial congruence between the CSs and the expert in monitoring snail populations. The CSs successfully identified the top 15 sites with the highest abundance of *Biomphalaria* spp. in over 84% of the matched reports. Moreover, we reported high values of the binary agreement for all snail genera (60-98%) across all the site types. These findings suggest that involvement of CSs has the potential to bridge the gap in snail data availability and the lack of snail experts [10,16,71], particularly in remote areas. But it can also significantly reduce logistical and financial costs (see below). Considering the increasing need for high-resolution data in precision mapping as the efforts shift from control to elimination of schistosomiasis [10,16], involving more people can address the issue of data scarcity. A notable example is the *Mosquito alert* project in Spain [21], which utilizes a mobile app allowing citizens to report sightings of the Asian tiger mosquito. This project demonstrated how a dense network of citizens can greatly enhance coverage compared to the traditional monitoring surveys that rely on a limited number of ovitraps. In our study, CSs collected high-resolution data both in terms of spatial coverage, encompassing 73 sites across an area of approximately 750km^2, and temporal coverage, sampling on a weekly basis for 20 months. Notably, ten of these sites were located in remote areas along the shores of Lake Albert in the highly endemic Ndaiga sub-county, making the resident CS the ideal person to regularly monitor them. Since abundance data (which was always lower than reported by the expert) is of less importance to guide snail control, we argue that citizen-generated snail data can guide targeted snail control.

However, it is important to note that in this study many locations without active schistosomiasis transmission but with high *Biomphalaria* spp. densities were found (e.g. the sites further away from the lake). Indeed, in order for schistosomiasis transmission to occur, other requirements like human water contact and open defecation need to be met [72,73]. Therefore, CSs also record the type and frequency of water-related activities they encounter when sampling. However, to prove actual schistosomiasis transmission, human or snail infection data is needed. Since this is outside the scope of our current citizen science approach, we recommend that citizen-driven snail monitoring should only be implemented in areas with known endemicity for schistosomiasis. In the future, one could envision the involvement of citizens in eDNA monitoring of schistosome DNA in waterbodies [74], as already done for the eDNA monitoring of birds and marine species [75,76]. One could even foresee the involvement of citizens in village-based mollusciciding as done in Cameroon [77], after snail detection or schistosome detection in the respective water contact sites.

Benefits beyond data quality

Citizen scientists offer a unique perspective on scientific and social issues that can complement or enrich those of professional scientists [30]. This fosters mutual learning between CSs and experts, allowing for subsequent exchange of knowledge and skills within their communities. Within the ATRAP project, the same CSs are also involved in community mobilisation for contextualised awareness campaigns. We have gained valuable insights from this citizen-led awareness campaigns where the CSs designed tailored messages on schistosomiasis prevention and control upon community input [78]. This led to diverse, contextualised communication approaches specific to each community, stimulating the knowledge uptake and moving away from a one-size-fits-all approach. Furthermore, Anyolitho et al. [79] reported existing myths and misconceptions regarding schistosomiasis in our study area, suggesting that bottom-up participatory approaches led by CSs can help address and reduce these misconceptions. Additionally, the process of data collection itself, which involves identifying snails associated with

schistosome transmission, raises awareness about snail-borne diseases in the area [78]. We observed that the support and demand for snail control by the CSs and their fellow community members increased during the project, and witnessed a multiplication effect by having CSs safely training their family members and friends in snail sampling and identification.

From a cost-benefit perspective, there are significant advantages to engaging CSs. It would require one expert at least 80 months to collect the data collected by the 24 CSs in 20 months. Moreover, given the size of our study area, weekly monitoring is simply not feasible for one person. For instance, monitoring the 73 sites on a weekly basis would require at least four experts, amounting to €178,640, which is about 7.6 times more expensive than working with CSs. This cost is based on the involvement of a PhD student as expert, so the total price would increase if governmental experts would be involved. The current cost for our sampling campaign is €23,475 including the initial non-recurring cost of buying equipment such as the smartphone, protective gear, and scoop net (for details, see *SI Appendix* Table S6).

In summary, we agree with Parrish et. [63] that the citizen science approach leads to increased ownership and sustainability of interventions, while providing opportunities for citizen involvement in scientific endeavours in a cost-effective manner.

Limitations and recommendations

The time-based sampling was preferred in this study to the more accurate yet cumbersome quadrat sampling [80], as the latter would be time consuming for volunteers, difficult to validate remotely compared to time stamps in the former, and the increased time investment would potentially lower the motivation of the CSs [81]. Irrespective of this choice, snail sampling is challenging for several reasons as discussed before.

With respect to *Bulinus* spp., the snail intermediate hosts of urogenital schistosomiasis, we need to further assess the applicability of the citizen science approach to map their distribution. Whereas the CSs correctly identified the sites where these snails occur, the relatively low spatial distribution of *Bulinus*

snails resulted in few CSs monitoring the sites where they occurred, precluding an in-depth comparison between CSs and the expert.

This study was conducted in a confined geographical area with only 25 CSs actively involved. This approach can be scaled up to other areas that are endemic for schistosomiasis. Considerations like the complexity of the tasks and the ability of the CSs to learn through feedback and experience should be carefully planned in the project design. Parrish at al. [63] suggested that for complex assignments continuous expert participation is recommended to improve the data quality, which was also shown by our own study [34]. Finally, identifying and understanding the motivations of citizen scientists will further improve the design and execution of citizen science projects to ensure mutual benefits for all involved parties.

In conclusion, we demonstrate that citizen scientist-collected data on freshwater snail intermediate hosts of *Schistosoma* and *Fasciola* parasites can be used to map putative snail-borne disease transmission sites. Citizen scientists can effectively detect snail occupancy where snails are abundant, but their effectiveness decreases in sites with low snail abundance. For studies where the exact abundance of snails is needed, we recommend expert surveys or integrating both approaches. Nevertheless, our approach is fit for the purpose of guiding targeted snail control and increasing community awareness. By transferring the knowledge about snail-borne diseases among local community members, awareness and ownership increase, which is vital for the success of control and prevention strategies. We therefore argue that this inclusive, powerful and cost-effective approach can be more sustainable than top-down monitoring and intervention campaigns.

Acknowledgments

This research was financed by the Action Towards Reducing Aquatic snail-borne Parasitic diseases (ATRAP) project of the Development Cooperation program of the Royal Museum for Central Africa and Mbarara University of Science and Technology with support of the Directorate-General

Development Cooperation and Humanitarian Aid. We also greatly appreciate the contribution of all the citizen scientists without whom this study would not have been possible: Alinda Hassan, Chotum Friday, Atanasi Marisel, Opio Isingoma, Ategeka Augustine, Bahemuka Bob, Masereka Haruna, Sebakara Fobius, Nyamahunge Imelda, Businge Zabron, Tumusiime Janet, Nsenga David, Night Marygoret, Kyaligonza Noeline, Mwesige Robert, Barisigara Gard, Tusingwire Henry, Natabi Specioza, Nakingi Rose, Bamuturaki Charles, Kamukama Josias, Nuwagaba Emmanuel, Tweheyo Julius, Bahungirehe Crezestom, Ategeka Rogers, Unimu Hadijah and Fuwarinyo Richard. Support from the Health Departments through the Vector Control Units of Kagadi and Ntoroko district local governments is acknowledged.

Data availability: The data used in this paper is available upon request. The code for validation of the citizen scientist collected is available on GitHub is available at the link https://github.com/CiSciUganda/data_validation/blob/main/Automatic_KOBO.

REFERENCES

1. Mage C, Bourgne H, Toullieu J, Rondelaud D, Deyfuss G. *Fasciola hepatica* and *Paramphistomum daubneyi*: changes in prevalences of natural infections in cattle and in *Lymnaea truncatula* from central France over the past 12 years. Vet Res. 2002;33(5):439–47.
2. Morgan JAT, J DR, Snyder SD, Mkoji GM, Loker ES. *Schistosoma mansoni* and *Biomphalaria* : past history and future trends. Parasitology. 2001;123(7):211–228.
3. Rollinson D, Stothard JR, Southgate VR. Interactions between intermediate snail hosts of the genus *Bulinus* and schistosomes of the *Schistosoma haematobium* group. Parasitology. 2001;123(SUPPL.).
4. Gryseels B, Polman K, Clerinx J, Kestens L. Human schistosomiasis. Lancet. 2006;368:1106–18.
5. Kyu HH, Abate D, Abate KH, Abay SM, Abbafati C, Abbasi N, et al. Global, regional, and

national disability-adjusted life-years (DALYs) for 359 diseases and injuries and healthy life expectancy (HALE) for 195 countries and territories, 1990-2017: A systematic analysis for the Global Burden of Disease Study 2017. Lancet. 2018;392(10159):1859–922.

6. Joan N, Stephen M, Bashir M, Kiguli J, Orikiriza P, Bazira J, et al. Prevalence and Economic Impact of Bovine Fasciolosis at Kampala City Abattoir, Central Uganda. Br Microbiol Res J. 2015;7(3):109–17.

7. Kokaliaris C, Garba A, Matuska M, Bronzan RN, Colley DG, Dorkenoo AM, et al. Effect of preventive chemotherapy with praziquantel on schistosomiasis among school-aged children in sub-Saharan Africa: a spatiotemporal modelling study. Lancet Infect Dis. 2022 Jan 1;22(1):136–49.

8. World Health Organization. Schistosomiasis [Internet]. 2021. Available from: https://www.who.int/news-room/fact-sheets/detail/schistosomiasis

9. King CH, Bertsch D. Historical Perspective: Snail Control to Prevent Schistosomiasis. PLoS Negl Trop Dis. 2015;9(4):2–7.

10. Sokolow SH, Wood CL, Jones IJ, Lafferty KD, Kuris AM, Hsieh MH, et al. To Reduce the Global Burden of Human Schistosomiasis , Use ‘ Old Fashioned ’ Snail Control. Trends Parasitol. 2017;

11. Qian C, Zhang Y, Zhang X, Yuan C, Gao Z, Yuan H, et al. Effectiveness of the new integrated strategy to control the transmission of *Schistosoma japonicum* in China: A systematic review and meta-analysis. Parasite. 2018;25.

12. Lo NC, Gurarie D, Yoon N, Coulibaly JT, Bendavid E, Andrews JR, et al. Impact and cost-effectiveness of snail control to achieve disease control targets for schistosomiasis. Proc Natl Acad Sci U S A. 2018;115(4):E584–91.

13. Sokolow SH, Wood CL, Jones IJ, Swartz SJ, Lopez M, Hsieh MH, et al. Global Assessment of

Schistosomiasis Control Over the Past Century Shows Targeting the Snail Intermediate Host Works Best. PLoS Negl Trop Dis. 2016;10(7):1–19.

14. World Health Organization. Ending the neglect to attain the Sustainable Development Goals: a road map for neglected tropical diseases 2021–2030. Geneva; 2019.

15. Allan F, Ame SM, Tian-Bi Y-NT, Hofkin B V., Webster BL, Diakité NR, et al. Snail-Related Contributions from the Schistosomiasis Consortium for Operational Research and Evaluation Program Including Xenomonitoring, Focal Mollusciciding, Biological Control, and Modeling. Am J Trop Med Hyg. 2020;1–14.

16. Rollinson D, Knopp S, Levitz S, Stothard JR, Tchuenté LT, Garba A, et al. Time to set the agenda for schistosomiasis elimination. Acta Trop. 2013;128(2):423–40.

17. Ashepet MG, Jacobs L, Oudheusden M Van, Huyse T. Parasitology Forum Wicked Solution for Wicked Problems : Citizen Science for Vector-Borne Disease Control in Africa. Trends Parasitol [Internet]. 2020;37(2):93–6. Available from: https://doi.org/10.1016/j.pt.2020.10.004

18. Fraisl D, Hager G, Danielsen F, Hitchcock BC, Hulbert JM, Piera J, et al. Citizen science in environmental and ecological sciences. Nat Rev Methods Prim. 2022;2(26):1–20.

19. Vohland K, Land-Zandstra A, Ceccaroni L, Lemmens R, Perelló J, Ponti M, et al. What Is Citizen Science? The Challenges of Definition. The Science of Citizen Science. 2021. 1–12 p.

20. Pocock MJO, Roy HE, August T, Kuria A, Barasa F, Bett J, et al. Developing the global potential of citizen science: Assessing opportunities that benefit people, society and the environment in East Africa. J Appl Ecol. 2019;56(2):274–81.

21. Palmer JRB, Oltra A, Collantes F, Delgado JA, Lucientes J, Delacour S, et al. Citizen science provides a reliable and scalable tool to track disease-carrying mosquitoes. Nat Commun. 2017;8(1):1–12.

22. Hamer SA, Curtis-Robles R, Hamer GL. Contributions of citizen scientists to arthropod vector

data in. Curr Opin Insect Sci. 2018;28:98–104.
23. Eritja R, Ruiz-Arrondo I, Delacour-Estrella S, Schaffner F, Álvarez-Chachero J, Bengoa M, et al. First detection of Aedes japonicus in Spain: An unexpected finding triggered by citizen science. Parasites and Vectors. 2019;12(1):1–9.
24. Murindahabi MM, Hoseni A, Corné Vreugdenhil LC, van Vliet AJH, Umupfasoni J, Mutabazi A, et al. Citizen science for monitoring the spatial and temporal dynamics of malaria vectors in relation to environmental risk factors in Ruhuha, Rwanda. Malar J. 2021;20(1):1–18.
25. Nieto NC, Porter TW, Lowrey TJ, Martin L, Motyka PJ, Salked DJ. Using citizen science to describe the prevalence and distribution of tick bite and exposure to tick-borne diseases in the United States. PLoS One. 2018;17(1):1–14.
26. Cox ET, Philippoff J, Baumgartner E, Smith MC. Expert variability provides perspective on the strengths and weaknesses of citizen-driven intertidal monitoring program. Ecol Appl. 2016;22(4):1201–12.
27. Kosmala M, Wiggins A, Swanson A, Simmons B. Assessing data quality in citizen science. Front Ecol Environ. 2016;14(10):551–60.
28. Elbroch M, Mwampamba TH, Santos MJ, Zylberberg M, Liebenberg L, Minye J, et al. The Value, Limitations, and Challenges of Employing Local Experts in Conservation Research. Conserv Biol. 2011;25(6):1195–202.
29. Fritz S, See L, Carlson T, Haklay M (Muki), Oliver JL, Fraisl D, et al. Citizen science and the United Nations Sustainable Development Goals. Nat Sustain. 2019;2(10):922–30.
30. Lukyanenko R, Parsons J, Wiersma YF. Emerging problems of data quality in citizen science. Conserv Biol. 2016;30(3):447–9.
31. Van Eupen C, Maes D, Herremans M, Swinnen KRR, Somers B, Luca S. The impact of data quality filtering of opportunistic citizen science data on species distribution model performance.

Ecol Modell [Internet]. 2021;444(September 2020):109453. Available from: https://doi.org/10.1016/j.ecolmodel.2021.109453

32. Kennedy EB. When citizen science meets science policy. In: The rightful place of science: Citizen Science. Washington DC: Consortium for Science, Policy & Outcomes; 2016.

33. Hecker S, Haklay M, Bowser A, Makuch Z, Vogel J, Bonn A. Innovation in open science, society and policy – setting the agenda for citizen science. In: Citizen Science. 2018. p. 1–24.

34. Brees J, Huyse T, Tumusiime J, Kagoro-Rugunda G, Namirembe D, Mugabi F, et al. The potential of citizen-driven monitoring of freshwater snails in schistosomiasis research. Citiz Sci Theory Pract. 2021;6(1):1–13.

35. KoBoToolBox. KoBoToolbox | Data Collection Tools for Challenging Environments [Internet]. 2014 [cited 2020 Jun 2]. Available from: https://www.kobotoolbox.org/

36. Brown DS. Freshwater Snails of Africa and Their Medical Importance. 2nd ed. London: Taylor & Francis; 1994. 1–673 p.

37. Mandahl-Barth G. Key to the identification of east and central African freshwater snails of medical and veterinary importance. Bull World Health Organ. 1962;27:135–50.

38. Rabone M, Wiethase JH, Allan F, Gouvras AN, Pennance T, Hamidou AA, et al. Freshwater snails of biomedical importance in the Niger River Valley: Evidence of temporal and spatial patterns in abundance, distribution and infection with Schistosoma spp. Parasites and Vectors. 2019;12(1).

39. Trevethan R. Sensitivity, Specificity, and Predictive Values: Foundations, Pliabilities, and Pitfalls in Research and Practice. Front Public Heal. 2017;5(307):1–7.

40. Schweinberger M. Fixed- and Mixed-Effects Regression Models in R. Brisbane: The University of Queensland. 2022.

41. Ugwu CLJ, Zewotir TT. Using mixed effects logistic regression models for complex survey

data on malaria rapid diagnostic test results. Malar J. 2018;17(1):1–10.

42. Zuur AF, Ieno EN, Elphick CS. A protocol for data exploration to avoid common statistical problems. Methods Ecol Evol. 2010;1(1):3–14.

43. Baayen HR. Analyzing Linguistic Data: A practical introduction to statistics using R. New York: Cambridge University Press; 2008. 1–353 p.

44. Kendall MG. The Treatment of Ties in Ranking Problems. Biometrika. 1945;33(3):239–51.

45. Agresti A. Analysis of Ordinal Categorical Data. 2nd ed. Balding JD, Cressie ACN, Fitzmaurice GM, Johnstone MI, Molenberghs G, Scott WD, et al., editors. New Jersey: John Wiley & Sons; 2010.

46. Krippendorff K. ScholarlyCommons Computing Krippendorff ' s Alpha-Reliability Computing Krippendorff ' s Alpha-Reliability. 2011;

47. Hart AG, Nesbit R, Goodenough AE. Spatiotemporal Variation in House Spider Phenology at a National Scale Using Citizen Science. Arachnology. 2018;17(7):331–4.

48. Woolhouse MEJ. Population Biology of the Freshwater Snail *Biomphalaria pfeifferi* in the Zimbabwe Highveld. J Appl Ecol. 1992;29(3):687.

49. Dewitt TJ, Sih A, Hucko JA. Trait compensation and cospecialization in a freshwater snail: Size, shape and antipredator behaviour. Anim Behav. 1999;58(2):397–407.

50. Morgan E, Last V. The behaviour of *Bulinus africanus*: A circadian profile. Anim Behav. 1982;30(2):557–67.

51. Goffredo S, Pensa F, Neri P, Orlandi A, Gagliardi MS, Velardi A, et al. Unite research with what citizens do for fun: '"recreational monitoring"' of marine biodiversity. Ecol Appl. 2010;20(8):2170–87.

52. Perez-Saez J, Mande T, Ceperley N, Bertuzzo E, Mari L, Gatto M, et al. Hydrology and density feedbacks control the ecology of intermediate hosts of schistosomiasis across habitats in

seasonal climates. Proc Natl Acad Sci U S A. 2016;113(23):6427–32.
53. Grabas GP, Fiorino GE, Reinert A. Vegetation species richness is associated with daily water-level fluctuations in Lake Ontario coastal wetlands. J Great Lakes Res. 2019;45(4):805–10.
54. Woolhouse ME. Passive dispersal of Bulinus globosus. Ann Trop Med Parasitol. 1988;82(3):315–7.
55. Gallas BD, Chen W, Cole E, Ochs R, Petrick N, Pisano ED, et al. Impact of prevalence and case distribution in lab-based diagnostic imaging studies. J Med Imaging. 2019 Jan 21;6(1):015501.
56. Leeflang MGM, Rutjes WSA, Reitsma BJ, Hooft L, Bossuyt MMP. Variation of a test's sensitivity and specificity with disease prevalence. Can Med Assoc. 2013;185(11):537–44.
57. Falade MO, Otarigho B. Shell morphology of three medical important tropical freshwater pulmonate snails from five sites in South-Western Nigeria. Int J Zool Res. 2015;11(4):140–50.
58. Pretorius SJ. Some aspects of the morphology of Lymnaea natalensis Krauss (Mollusca: Basommatophora). North-West University (South Africa); 1963.
59. Bancks N, North EA, Johnson GR. An analysis of agreement between volunteer- And researcher-collected urban tree inventory data. Arboric Urban For. 2018;44(2):73–86.
60. Galloway AWE, Tudor MT, Haegen WM Vander. The Reliability of Citizen Science: A Case Study of Oregon White Oak Stand Surveys. Wildl Soc Bull. 2006;34(5):1425–9.
61. Roman LA, Scharenbroch BC, Östberg JPA, Mueller LS, Henning JG, Koeser AK, et al. Data quality in citizen science urban tree inventories. Urban For Urban Green. 2017;22:124–35.
62. Engel SR, Voshell JR. Volunteer biological monitoring: Can it accurately assess the ecological condition of streams? Am Entomol. 2002;48(3):164–77.
63. Parrish JK, Burgess H, Weltzin JF, Fortson L, Wiggins A, Simmons B. Exposing the Science in Citizen Science: Fitness to Purpose and Intentional Design. Integr Comp Biol. 2018;58(1):150–60.

64. Camilleri A, Gatt A, Formosa C. Inter-rater reliability of four validated diabetic foot ulcer classification systems. J Tissue Viability. 2020;29(4):284–90.

65. Terzis A, Klinger A, Seegmüller J, Sauerbier M. Inter-rater reliability of magnetic resonance imaging in comparison to computed tomography and wrist arthroscopy in slac and snac wrist. J Clin Med. 2021;10(16).

66. Schilling AT, Ehresman J, Pennington Z, Cottrill E, Feghali J, Ahmed AK, et al. Interrater and Intrarater Reliability of the Vertebral Bone Quality Score. World Neurosurg. 2021;154:e277–82.

67. Crall AW, Jordan R, Holfelder K, Newman GJ, Graham J, Waller DM. The impacts of an invasive species citizen science training program on participant attitudes, behavior, and science literacy. Public Underst Sci. 2013;22(6):745–64.

68. Newman G, Chandler M, Clyde M, McGreavy B, Haklay M, Ballard H, et al. Leveraging the power of place in citizen science for effective conservation decision making. Biol Conserv. 2017;208:55–64.

69. Berland A, Roman LA, Vogt J. Can field crews telecommute? Varied data quality from citizen science tree inventories conducted using street-level imagery. Forests. 2019;10(4):1–18.

70. Palacin V, Gilbert S, Orchard S, Eaton A, Ferrario MA, Happonen A. Drivers of participation in digital citizen science: Case studies on Järviwiki and safecast. Citiz Sci Theory Pract. 2020;5(1):1–20.

71. Shiff C. Why reinvent the wheel? Lessons in schistosomiasis control from the past. PLoS Negl Trop Dis. 2017;11(10):1–6.

72. Exum NG, Kibira SPS, Ssenyonga R, Nobili J, Shannon AK, Ssempebwa JC, et al. The prevalence of schistosomiasis in Uganda : A nationally representative population estimate to inform control programs and water and sanitation interventions. PLoS Negl Trop Dis.

2019;13(8):1–21.

73. Utzinger J, N'Goran EK, Caffrey CR, Keiser J. From innovation to application: Social-ecological context, diagnostics, drugs and integrated control of schistosomiasis. Acta Trop. 2011;120(SUPPL. 1).

74. Sengupta ME, Lynggaard C, Mukaratirwa S, Vennervald BJ, Stensgaard AS. Environmental DNA in human and veterinary parasitology - Current applications and future prospects for monitoring and control. Food Waterborne Parasitol. 2022;29(February):e00183.

75. Tøttrup AP, Svenningsen L, Rytter M, Lillemark MR, Møller P, Knudsen SW. Citizens in the Lab: Performance and Validation of eDNA Results. Citiz Sci Theory Pract. 2021;6(1):1–12.

76. Biggs J, Ewald N, Valentini A, Gaboriaud C, Dejean T, Griffiths RA, et al. Using eDNA to develop a national citizen science-based monitoring programme for the great crested newt (*Triturus cristatus*). Biol Conserv. 2015;183:19–28.

77. Greer GJ, Tchounwou PB, Takougang I, Monkiedje A. Field tests of a village-based mollusciciding programme for the control of snail hosts of human schistosomes in Cameroon. Trop Med Int Heal. 1996;1(3):320–7.

78. Anyolitho MK, Huyse T, Masquillier C, Nyakato VN, Poels K. Combining citizen science and other participatory approaches to implement behaviour change interventions: (How) Does this work for schistosomiasis prevention among endemic communities in western Uganda. Prep.

79. Anyolitho MK, Poels K, Huyse T, Tumusiime J, Mugabi F, Tolo CU, et al. Knowledge, attitudes, and practices regarding schistosomiasis infection and prevention: A mixed-methods study among endemic communities of western Uganda. PLoS Negl Trop Dis. 2022;16(2):1–21.

80. Perez-Saez J, Mande T, Zongo D, Rinaldo A. Comparative analysis of time-based and quadrat sampling in seasonal population dynamics of intermediate hosts of human schistosomes. PLoS Negl Trop Dis. 2019;13(12):e0007938.

81. Sprinks J, Wardlaw J, Houghton R, Bamford S, Morley J. Task Workflow Design and its impact on performance and volunteers' subjective preference in Virtual Citizen Science. Int J Hum Comput Stud. 2017;104(March):50–63.

Supporting information captions

***SI Appendix* Fig S1.** Site types used in the grouping of data collected by Citizen Scientists (CS) and the expert. Springs are underground water sources trapped by concrete walls to direct water through a metallic pipe with continuously flowing water, accessed by the communities. Stream sites are water contact sites with flowing surface water. Lake sites on Lake Albert were highly used water contact sites within fishing villages in Ndaiga Sub County and Ntoroko Town Council, on the South-Eastern side of the lake. Wetland sites consist of flooded areas for most of the year mostly characterised by rooted aquatic vegetation.

***SI Appendix* Fig S2.** The effect of expert snail removal and sampling date difference with the CS for *Biomphalaria*, *Bulinus* and *Radix* snails at the different site types. We explored the potential impact of snail removal without replacement by the expert. It was hypothesised that if snail removal impacted the extent of agreement between the expert and CS data, agreement would be higher if the CS sampled before the expert, more so on same date. Similarly, the effect of snail removal by the expert was expected to decrease with time due to migration. Thus, "Same" stands for the expert and CS having sampled a site on the same day, "Before" when the expert sampled before the CS and "After" when the expert sampled after the CS. If the sampling date difference was between one and three, it was considered "Close" (grey error bars) and if the difference was between four and six days, it was considered "Apart" (black error bars). Except for *Biomphalaria*, the difference in sampling days did not explain significant agreement/disagreement in snail presence/absence. Across all the genera and site types, the effect of snail removal by the expert was not significant (X^2 (2) = (2.3, 2.1 & 2.5) $p > 0.05$, for *Biomphalaria, Bulinus* and *Radix* respectively) when the expert sampled before the CS.

***SI Appendix* Fig S3.** Observed probability of binary agreement (presence/absence) of *Biomphalaria* (A), *Bulinus* (B) and *Radix* (C) compared in the period when the expert removed snails from a site without replacement (16 months) with when the expert replaced the snails (4 months). For the three genera, the probabilities of agreement are statistically different with $p<0.05$. If snail removal changes snail population dynamics, the probability of agreement was expected to be lower when there was snail removal. However, except for *Radix* snails, the probability of was higher in the period when there was snail removal, contrary to our expectation. There was no clear pattern in agreement as a result of snail removal implying no clear influence of the snail removal by the expert on agreement in snail presence/absence.

***SI Appendix* Fig S4.** Structure of the nested random effect implemented in the linear mixed-effect model. ID is the CS ID, loc is the sampling site/location.

***SI Appendix* Fig S5.** Map of the study area showing total abundance (expert data 06/2020 – 04/2022) of the studied snail genera in each water contact site, black points indicate locations without snails.

***SI Appendix* Fig S6**. Observed probability of binary agreement with time between the citizen scientists and the expert in *Radix* sp. presence/absence with time.

***SI Appendix* Fig S7.** Observed variation in the degree of agreement of each citizen scientists with the expert in detection of presence/absence of *Biomphalaria*, *Bulinus* and *Radix* snails. We observed variations in agreement for the same citizen scientist when sampling different site types. The citizen scientist IDs were sorted from lowest to highest mean agreement with the expert for each snail genus.

SI Appendix Fig S8. Analysis including cumulative abundance reported by the citizen scientists (dotted line) and the expert (full lines) per month (left panel). The differences between the graphs (Fig 3A – main text) are due to the number of points that are being considered (1037 points for each snail genera, in contrast with 911 paired reports on Biomphalaria spp., 900 on Bulinus spp. and 905 on Radix sp. from

the original analysis) because originally, we only considered points that were apart +/- 7 days. The false positives increased, but also the agreement (TP + TN) increased for Radix by around 10%.

***SI Appendix* Fig S9.** Values of consistency and numerical per time step (month) for all the snail genera studied.

***SI Appendix* Table S1.** Variables used in the comparison of citizen scientists' and expert collected snail data.

***SI Appendix* Table S2.** *Biomphalaria, Bulinus* and *Radix* predictors of agreement in snail presence/absence between the citizen scientists and the expert, and their metrics. Ref. stands for reference, CI for confidence interval, and significant values are in bold.

***SI Appendix* Table S3:** Generalized linear mixed models output – case A

***SI Appendix* Table S4:** Generalized linear mixed models output – case B

***SI Appendix* Table S5**. Analysis including cumulative abundance per month (CS) / Abundance

***SI Appendix* Table S6.** Summary of cost of data collection by the citizen scientist compared to the expert. For the expert, two scenarios are considered: when one expert samples each site once a month as in our approach, and if the expert were to sample each site four times a month like the citizen scientists in this study.

Click here to access/download
Supporting Information
Supplementary Information_Tumusiime et al.docx
