# Supplementary material for: Citizens can help to map putative transmission sites for snail-borne diseases
Source: PLoS Negl Trop Dis. 2024 Apr 4;18(4):e0012062. doi: 10.1371/journal.pntd.0012062 (PMC11020946; doi:10.1371/journal.pntd.0012062)
Supplement: Attachment — Submitted filename: Rebuttal Letter_PNTD-D-23-00886-Version Feb 06.docx [file pntd.0012062.s018.docx]

Manuscript number: PNTD-D-23-00886

**Citizens can help to map putative transmission sites for snail-borne diseases**

Authors: *Julius Tumusiime a,b,1*, Noelia Valderrama Bhraunxs c,d,1, Grace Kagoro-Rugunda a, Daisy Namirembe a, Christian Albrecht b,c, Ronald Twongyirwe e, Casim Umba Tolo a, Liesbet Jacobs d,e & Tine Huyse c*

Dear Editor,

Thank you for refereeing our paper. We are grateful for the insightful comments and the constructed feedback provided. Below, we address carefully each one of them, incorporating the necessary revisions to our manuscript. We are convinced that the new version meets the required standards.

Best wishes,

Julius Tumusiime, on behalf of all the co-authors

| **Reviewer’s comment** | **Response** |
| --- | --- |
| **REVIEWER 1** | |
| 1) Ethics: There is no mention of ethics review or approval for the study. It might be possible that the Ugandan authorities may not have required it since they involved malacological data. | This has been addressed with a sub section ‘**Ethical considerations’** added in the methods section (line 185-193).  “*This study was approved by the Mbarara University of Science and Technology Ethical Review Committee with reference number MUREC 1/7, and by the Uganda National Council of Science and Technology under the reference number NS148ES. In addition, written informed consent was sought from the citizen scientists who participated in this study. The extent of disclosure of the information they provided and their facilitation were fully discussed and formalised in a memorandum of understanding before the commencement of the data collection process. The memorandum of understanding was renewed annually following a refresher training to allow the CSs to share their experiences and expectations*.” |
| 2) There is a small typo in Fig 3. The graph is divided between A,B,C and D sections. The legend only lists A-C, although the text covers all 4 areas | Corrected. We have included the letter D which was missing. |
| 3) The legends for Figures 1 and 3 will require to specify the sources of images to ensure no copyrights are vulnerated | Corrected. We included Biorender and Open streets map as source of the snail icons and map, respectively. For the snail icons, the Biorender licence numbers are WS268PLCB9, ON268PKGNM and XU268PL5IV for *Radix*, *Biomphalaria* and *Bulinus* respectively. |
| 4) I would recommend the authors to provide a minimum of information about site selection process and operational definition of water points visited. Whilst these subjects can be consulted in another reference, it'd help the reader to have a broader understanding of the study if these key points of the paper are stated explicitly in the text | Thank you for your recommendation. We have included the following text in the methods section to provide more context in the selection of the study sites and the operational definition of the water contact point (lines 120-132):  *“Briefly, field exploration visits were conducted to identify candidate water contact sites for the monitoring programme in the endemic Lake Albert region. Topographic gradients (lake or upland), hydrological gradients (small streams, large rivers, artificial springs, wetlands, lake) and human water contact intensity (very low to very high) were considered. Community knowledge about schistosomiasis, implied from presence of a routine MDA programme, guided the selection of the study sub counties, to motivate the participants to intervene in a known problem to them. This resulted in 77 study water contact sites in the selected eight sub counties in Kagadi and Ntoroko districts. A water contact site was defined as a delineated part of a water body for human activities such as fishing, bathing, swimming etc., for at least part of the year [34]. For consistence and comparison, a 10-metre stretch was delineated at each site while sites smaller than approximately 100 m² were considered entirely. When Lake Albert experienced flooding and recession of the water levels during this study period, the new sampling locations (15 affected sites) were agreed upon between the expert and respective citizen scientist.* “ |
| 5) The project depended on a substantive commitment by citizen scientists. It would be important to highlight that monthly transportation and mobile data costs were covered by the project (if not stated, the reader may have an incorrect interpretation of the demands on residents). It would be useful to know as well if any measures were adopted to keep volunteers' motivation high throughout the study. | The following information is now explicitly outlined in line 134-136: “*Apart from a mobile phone, a backpack and t-shirt with the ATRAP logo, the CSs also received monthly financial compensation to cover transportation costs and data bundles”.*    In lines 141-147, we have included the following phrases to provide insights on measures adopted to motivate the CSs: “*Furthermore, a WhatsApp group was created, facilitating communication between citizen scientists and experts. This platform streamlined interaction and enhanced collaboration. Moreover, the refresher trainings and awareness campaigns annually organised contributed to the citizens motivation. In addition, direct communication lines were open through phone calls with the project administrative assistant and the research team members. This enabled quick problem-solving whenever technical (e.g. with the smartphones) and personal project-related challenges arose.”* |
| 6) Concerning ethics, it is recommended that the authors provide a statement indicating how this subject was approached. If no ethics approval was required by Uganda's MoH or what additional institutional reviews the project went through. | Omission of the ethical consideration statement was a major oversight on our part. However, we followed ethical guidelines at the local institutional and national levels as now indicated under the subheading “**Ethical considerations**” as elaborated in the review comment 1 above. |
| 7) It would be recommended expand the section "Can citizens help to map putative schistosomiasis transmission sites to guide targeted snail control?" with information more directly related to schistosomiasis. That is, addressing more directly how existing or prospective snail control project designs / approaches can be directly informed by the CS evidence, and to what extent the quality of the evidence gathered is sufficient to that effect. | We agree with this suggestion. However, since we were testing the citizen science approach for the first time in snail monitoring, we cautiously discuss the basics such as snail monitoring (presence/absence and abundance) by the citizens. We think there is still room for further engagement of the citizens in activities such as snail testing and snail control to be able to provide schistosomiasis - specific recommendations supported by the generated data. These activities/initiatives are foreseen in the following phase of the project.  We revised the respective section and added the following lines (585-595):  *“Moreover, we reported high values of the binary agreement for Biomphalaria spp. (76%) across all the site types, and a low number of false negatives* *(15.4%) (see above). The latter is important since false negatives would lead to refraining from snail control in potential transmission sites. These findings, together with the similar trend in relative snail abundance over time generated from expert and CS data, suggest that involvement of CSs has the potential to bridge the gap in snail data availability and the lack of snail experts [14,20,75]. It could generate the data needed by the national Vector Control Division for planning targeted snail control interventions, and meet the increasing need for high-resolution data in precision mapping as the efforts shift from control to elimination of schistosomiasis [14,20]. Involving local citizens could increase the spatial coverage, facilitate access to remote areas and decrease the monitoring costs (see below).”* |
| **REVIEWER 2** | |
| 8) This is not the correct way of writing sub-Saharan Africa. | Corrected |
| 9) Something about this sentence needs to be fixed. The contrast and presence of the word hinders after data makes it difficult to understand the contrast being suggested. | In lines 19-21, we have changed the sentence to: “*The World Health Organization recommends complementing drug treatment with snail control and community involvement for disease elimination, but there is a general lack of snail experts and hence snail distribution data*”. |
| 10) Several sections have are not supported by literature when its evident that these ideas have been taken from somewhere. This needs to be fixed. | Noted and addressed accordingly. We tried to back up our statements with the respective literature and added the following new citations: [1], [2], [3] [9], [83]. |
| 11) Fixed this throughout the manuscript. Its supposed to be sub-Saharan Africa not Sub-Saharan..... | Corrected. |
| 12) Line 67-68: Can this be supported by literature or is it the authors won information? | A citation of the source has been included [6](Mehmood et al., 2017) |
| 13) Line 96: A sentence or paragraph can’t start with this word unless it contrasting an immediate sentence, in the same paragraph. | Addressed. |
| 14) Line 108-109: In its current format, this would best fit in the methods section than introduction. As such, it needs to be recast to be fir for the background. | This is well noted and adjusted according by recasting it with the aim statement as follows:  *“To assess the quality of the collected data, the same sites were also sampled by an expert malacologist. Our aim now is to compare both datasets and assess the extent of agreement in (i) snail occurrence….”* |
| 15) Line 110-111: What’s the difference between this aim and the aim in line 112? | We first outlined the specific aims of the article while the following statement illustrates the overall aim. We adjusted the order in lines 109-113 to avoid confusion:  “*Our overall aim was to explore the potential of citizen science in mapping putative transmission sites for guiding targeted snail control by assessing the extent and type of observational biases. Specifically, we compared both datasets and assess the extent of agreement in (i) snail occurrence (presence/absence) - referred to as binary agreement, and (ii) snail abundance - evaluated as consistency and numerical agreement*”. |
| 16) Line 123: Collected not reported. | This has been rectified by replacing “reported” with “collected”. |
| 17) Line 128-129: Was this protocol submitted as part of the supplementary files? if not why? | The protocol has now been added as part of the supplementary files (SI Appendix 1) as indicated in line 149. |
| 18) Line 136-137: So the infection status of the snails was not assessed? Returning of infected snails into the water which sounds like this was capture and return, perpetuates the risks of infection of schistosomiasis or fasciolosis? How then do the authors reconcile this considering the introduction section talks about achieving the WHO road map target of eliminating schistosomiasis by 2023? | Infection status of the snails was determined using the snail samples collected by the expert on a monthly basis, so these snails were not returned. Regarding the returning of the snails collected by the citizens we had several discussions amongst our team members, and with the respective communities and citizen scientists. Since a large part of our study area is not endemic for schistosomiasis (upland area), a weekly removal of snail species would impact the aquatic ecosystem. Removing snails in the endemic areas only would preclude any meaningful comparison among different site types. Secondly, the citizen science approach had not been applied in snail monitoring before. Therefore, we needed data on how well the citizens can detect spatial and temporal trends in snail abundance. Snail removal on a weekly basis would introduce a bias in the data complicating the assessment of the natural trends and the effectiveness of the citizen science approach.  In the final four months of the study, the expert also returned the snails to the water to test whether we could detect any bias potentially introduced by snail removal on agreement/disagreement between the raters. As was explained in the manuscript in line 191-193, no effect could be detected. This also suggests that snail removal at this level of intensity and frequency would not contribute to snail control.  We changed the following statement in line 156-159:  “*All the collected snails were returned to the site after recording the information to minimise the overall environmental impact as well as the snail population dynamics in particularly, which we aimed to detect through citizen science sampling*.” |
| 19) Line 146-147: Was the marking of presence/absence based on snail availability or genus availability?   In line 125, ”.."focusing on species belonging to *Biomphalaria, Bulinus* and/or *Rad”x*." was this part of the training? | We hope to have interpreted the comment correctly and assume that the reviewer meant whether we identified the snails based on genus or species level. The answer is, we identified the three genera of interest (*Biomphalaria*, *Bulinus* and *Radix*) but pooled all other groups without further classification.  Yes, we focused on the three genera in the training to ensure that the citizens were able to identify them. While we provided additional summarised guides to enable them to identify a few other genera that could easily be confused with the species of concern, we did not emphasise their classification. We anticipated that overloading the CSs with information could result in confusion. Despite this approach, it’s noteworthy that all the snails encountered, including those outside the three specific genera, were almost always submitted by the CS under the correct designated labels of *Biomphalaria, Bulinus, Radix* or pool. |
| 20) Line 154-156: So the CS visited the sites on a weekly basis while the expert visited on a monthly basis? | Yes. It was not logistically possible to have an expert visiting all the 76 sites on weekly basis like the CSs.  We have rephrased the statement to remove any ambiguity:  *'Therefore, a trained malacologist (JT) (hereafter referred to as the ‘expert’) visited monthly the same sites that were monitored by the CSs weekly, following the same standardised snail collection protocol.’* |
| 21) Line 165-167: And why not those snails collected by the CS? Dont you think training the CS how to shed snails would be helpful and identifying those that are infected and remove them from the sites to reduce the risk of infection? | This exact suggestion has also been considered by the ATRAP team. However - in the current set up and context - safety during these shedding experiments cannot be ensured - leaving a substantial risk for infection. Additionally, this would require us to equip all citizen scientists with microscopes, which was not feasible. We therefore opted against this at the time being. |
| 22) Line 174: Refer to line 155-156 the Css visited on a weekly basis thus, their presence/absence was for weekly returns. The expert visited on a monthly basis thus his presence or absence was a onetime point data. How then was data matched in space and time? | For each expert report at a specific location, we considered the corresponding citizen report for the same location that was close to that timepoint, being within a range of seven days before or after the date of expert sampling. We matched both in time and space in order to have meaningful comparisons, so we only included a subset of citizen data to ensure alignment with the expert's data, covering the same location and period. This has been explained in the Methods section in lines 198-201. |
| 23) Line 188-190: Use this to answer the query on the results. | We agree with this suggestion. Indeed, this has been alluded to in the introductory section of the discussion in which we point out the limitations of the ‘ground-truth’ data collected by the expert. For emphasis, we have now included ‘*like false positives/negatives*’ in line 461-462 to make it more explicit. |
| 24) Line 233-234: Did this also involve taking the nearest count dates between the two sampling periods? If so, wasnt time of sampling for each (expert and CS) a factor to consider in the model as it may have had an influence on abundance? (mortality due to known and unknown factors influencing abundance) | Yes, we assessed the closest count dates between the two sampling periods. While we tested the sampling time as a predictor for snail abundance (see SI Appendix Table S3-S4), we did not find a significant relationship. However, the difference in dates did impact the agreement on presence/absence between experts and citizens for *Biomphalaria*. We would like to refer to Fig.2 and to lines 365 -368. |
| 25) Line 293: What are data reports? Snails or what? | We call them data report because they reported about snails (being present or absent) and other variables such as human activities and water chemistry parameters which are out of the scope of this manuscript. We think calling the submitted reports snail reports may be misleading.  We have rephrased this as “snail and ancillary data reports” to make it clearer. |
| 26) Line 293-295: How does this reconcile with what is in line 188-190? Why were the dats filtered if the earlier texts suggests that the nearest date between the expert and Cs sampling was taken? Furthermore, why were the dates filtered out as this is a randon variable that may have influence on snail counts? | There was no further filtering based on date. Basically, this statement emphasises the same point. Only the closest time points were considered (≤ 7 days). In addition, data reports with errors were also excluded to have a final dataset for comparison containing data from the expert and the CSs.  Thus, date differences ranging between 0 to 7 was still maintained and used as a variable to analyse agreement in snail presence/absence, and snail counts. |
| 27) Line 305-306: I hope there is an explanation for this difference in the discussion. | Yes, the differences were explained in the discussion section under the subheading “**Can citizens correctly monitor snail presence and absence?”** |
| 28) Line 308: past tense not present tense. | Thank for pointing it out. It has been corrected from ‘exhibit’ to “exhibited” |
| 29) Line 322 (Fig 2.): This need to be aligned properly as they may be confusing | We have included letter codes previously missed for each panel to ease understanding of the figure. |
| 30) Line 360-361: Is this surprising? if so, why? if not, why? | This was explained in line 567 in the discussion section:  *A plausible explanation for the differences in snail abundance between CSs and the expert is the task difficulty. Determining the presence or absence of a particular snail genus requires relatively low skill, which CSs would already acquire after the initial malacological training. However, obtaining reliable abundance data comparable to the ground-truth data (expert data) requires that, in addition to identifying the potential microhabitats of the snails at the sampling sites, a vigorous and continuous sampling for 30 minutes should follow, which demands considerable physical effort and dedication. In the latter, individual factors such as motivation or skill, could contribute to the observed variations in performance among CSs (see SI Appendix Fig S7). The observed increase in agreement in the ranking of high abundance sites over time supports the hypothesis of task difficulty, indicating that CSs improve their performance with more practice and experience, but also after the refresher training that stressed the importance of correct sampling (Fig 3D).* |
| 31) Line 403 (Discussion): The first paragraph of the discussion should highlight the significant findings and or strange findings which then can be discussed in depth in the later paragraph.   The initial paragraph of this paper doesn't show that and would need recasting extensively. | We agree with the referee that this usually how it is done, but we exceptionally and purposively chose for another strategy, as for this particular study, it is crucial to first explain the ‘fitness-for-purpose” concept, as this guides the entire discussion on data quality. Thus, while we appreciate the suggestion to highlight the key findings at the beginning of the discussion, we strongly prefer this structure in which this ‘unusual’ approach in snail-borne disease monitoring is contextualised. |
| 32) Line 426-428: These two fundamental questions need to be aligned with the study aim to which the results point to. | Fortunately, these two questions are directly translated from the aims of the study as indicated in line 111. |
| **REVIEWER 3** | |
| 33) Line 25: change “while” with “and”. | Line 36, addressed. |
| 34) Line 147: Altogether, reporting errors due to snail misclassification, inaccurate sampling location, and incorrect and incorrect scoop time make less than 2.5% of total reports (line 151). Personally, I think that snail classification is very different from inaccurate sampling location, and incorrect and incorrect scoop time, as it tells us also how good are citizen scientists in classifying snails (and it looks like they are very good), I am curious to know the fraction of snail misclassifications, and whether misclassification occurred especially between Bulinus and Radix. | This is a noteworthy observation. The majority of misidentifications were associated with *Bulinus* and *Radix*, comprising 111 and 80 reports, respectively, out of a total of 5,398 reports (number of reports until 30/06/2022 without filtering only the ones matching dates with the expert). For *Biomphalaria*, this value is lower at 41 reports. However, in general, snail misidentification is less than 0.02% for all genera.  We added the following statemen in lines 324-326 to bring attention to this comment:  *‘The expert collected a total of 1,382 snail and ancillary data reports, while the CSs collected 5,398 reports until 30/06/2022. From all the reports by the CSs, snail misidentification was less than 0.02% for all genera,* *consisting of 111 reports on Bulinus, 80 on Radix, and 41 on Biomphalaria.’*  Concerning misclassifications between *Bulinus* and *Radix*, these errors were particularly prevalent in the first months of sampling. However, citizens gradually improved their ability to differentiate them, primarily by focusing on the side of the snail opening. There were instances where *Melanoides* was confused with *Radix* as a consequence of the previous advice. Following effective communication with citizens, these mistakes, while not entirely eliminated, were considerably reduced. We acknowledge that some residuals mistakes might be attributed to occasional distraction during the sampling process.  For more details on snail classification accuracy and reduction in errors with time and following feedback, we refer you to a paper by Brees et al (2021) <https://doi.org/10.5334/cstp.388>. |
| 35) Line 167-169: <<in the last in the last four months of snail sampling, all snails were returned to the site after enumeration…>>: I am curios to now whether there is knowledge of prevalence of infection in the human population, and whether snails were sampled at transmission sites or elsewhere – did the authors ponder the risk of returning potentially infected snails in the water body where human infection could potentially occur? | Indeed, this is an important question and we did consider this carefully, and like to refer to our answer above.  Human schistosomiasis infection in the vicinity of our study area is reported among shore dwelling communities to be >50% by Exum et al (2019), although this study did not include our study area itself. Our snail infection data (not included in this study), confirms this pattern, as we only found infected snails in the lake sites, not upland. |
| 36) Line 191 and following. The authors clearly define sensitivity. Below in results they use specificity, but, unless I missed, they didn’t define it in this paragraph – if this is the case, I wonder whether they might want to provide the definition year. | We provided information about sensitivity which we defined indeed in the manuscript. We did not consider specificity as an important aspect of our discussion for snail occupancy, because true negatives do not inform much about the disease risk. |
| 37) Line 202-203: As for the nested random effects, I suggest to make explicit reference to the figure in the supplementary information “SI Appendix Fig S4”, which is really clear. | Thank you for your suggestion, this has been addressed. |
| 38) Line 257-258: maybe I am picky but, in the formulas eq. 4, the authors use “n.p” and “n.q” (number of snails sampled by CSs and experts), where here they define plain “p” and “q” – should the definition of “n.p” and “n.q” be explicitly reported in the text? | Indeed, this has been modified and the terms n.p and n.q defined in the lines 288-290. |
| 39) Line 275: consider removing “and 1 perfect numerical agreement” as you have already said so at line 272. | Addressed. |
| 40) Fig. 2: I think the letters A, B… E to identify the five panels are missing. | That was an oversight. Thank you for pointing this out, it has now been corrected. |
| 41) As additional minor point, RE:Line2 121-127: I looked at Brees et al. 2021, but I do not think that it is all that clear how data submission via the Kobo toolbox occurred, I was wondering whether the authors could add an appendix in the supplementary information with a slightly more expanded explanation or description of the Kobo app/interface to store the data, whether each CS typed in the data at the transmission site, etc.. | We added the protocol with the questions in the Supplementary Appendix 1. There is possible to see which kind of data the CSs submitted. |
